# Alterations in sorting and secretion of hepatic apoA5 induce hypertriglyceridemia due to short-term use of olanzapine

**DOI:** 10.3389/fphar.2022.935362

**Published:** 2022-08-12

**Authors:** Piao-Piao Huang, Wen-Qiang Zhu, Jing-Mei Xiao, Yi-Qi Zhang, Rong Li, Yang Yang, Li Shen, Fei Luo, Wen Dai, Ping-An Lian, Ya-Xin Tang, Juan-Li Ran, Xian-Sheng Huang

**Affiliations:** ^1^ Department of Cardiovascular Medicine, The Second Xiangya Hospital, Central South University, Changsha, Hunan, China; ^2^ Research Institute of Blood Lipid and Atherosclerosis, The Second Xiangya Hospital, Central South University, Changsha, Hunan, China; ^3^ National Clinical Research Center for Mental Disorders, and Department of Psychiatry, The Second Xiangya Hospital of Central South University, Changsha, Hunan, China; ^4^ Department of Critical Care Medicine, The First Affiliated Hospital, School of Medicine, Zhejiang University, Hangzhou, China; ^5^ Department of Stomatology, The Second Xiangya Hospital, Central South University, Changsha, Hunan, China; ^6^ Department of Molecular Genetics, University of Texas Southwestern Medical Center at Dallas, Dallas, TX, United States; ^7^ Department of Medicine, Columbia University Medical Center, New York, NY, United States

**Keywords:** olanzapine, hypertriglyceridemia, apolipoprotein A5, sorting, secretion

## Abstract

Long-term use of olanzapine, an antipsychotic drug, induces hypertriglyceridemia, resulting in a higher risk of cardiovascular disease. However, the effects and underlying mechanisms of short-term use of olanzapine on circulating triglyceride levels remain poorly understood. Here, the role of apolipoprotein A5 (apoA5), a regulator of triglyceride metabolism, was investigated in olanzapine-induced hypertriglyceridemia. Our multi-center clinical study recruited 36 schizophrenia patients who received short-term (8 weeks) of olanzapine. Besides, female C57BL/6J mice were treated with olanzapine (3 mg/kg/day versus 6 mg/kg/day) for 6 weeks. We demonstrated that short-term use of olanzapine increased plasma triglyceride and decreased plasma apoA5 levels in the patients and mice, with a negative correlation between the two factors. However, no obesity was observed in the patients and mice. Interestingly, olanzapine increased hepatic apoA5 protein in the mice, without significant changes in hepatic *Apoa5* mRNA. Consistently, *in vitro* studies indicated that olanzapine increased medium triglyceride levels and decreased medium apoA5 levels in a dose-dependent manner in human HepG2 cells and primary mouse hepatocytes. Whereas the olanzapine treatment increased hepatic apoA5 protein *in vitro*, without effects on hepatic *APOA5* mRNA. Of note, olanzapine increased the co-localization between apoA5 protein and accumulated lipid droplets in hepatocytes, as opposed to at the hepatocellular plasma membrane, in mouse liver as demonstrated by fluorescence staining. Therefore, our study indicated that short-term use of olanzapine induced hypertriglyceridemia due to defects of sorting and secretion of hepatic apoA5.

## 1 Introduction

Olanzapine, an atypical antipsychotic drug, is among the most effective therapies for schizophrenia patients because of its broad-spectrum efficacy and low risk of extrapyramidal symptoms (Lieberman et al., 2005). However, long-term use of olanzapine in schizophrenia patients leads to hypertriglyceridemia that increases cardiovascular risk (Vancampfort et al., 2015). It is well acknowledged that obesity, due to long-term use of olanzapine-associated changes of diet (high-fat diet) and lifestyle (sedentary lifestyle and less exercise) in schizophrenia patients, contributes to lipid disturbance (Correll et al., 2009; Nielsen et al., 2010; De Hert et al., 2011). Traditionally, therefore, the effects of olanzapine on lipid disturbance are considered indirect, *i.e.,* long-term use of olanzapine induces obesity and consequently hyperlipidemia. To date, the effects of short-term use of olanzapine on lipid metabolism remain unknown. Besides, it is also unclear whether olanzapine-induced hypertriglyceridemia results from the effects of this drug on lipid-related mediators. Therefore, this study aimed to determine the effects and related mechanism of short-term use of olanzapine on plasma triglyceride levels.

In this study, the role of apolipoprotein A5 (apoA5) in the development of olanzapine-induced hypertriglyceridemia was investigated. ApoA5 is a negative regulator of plasma triglyceride levels, that is, specifically synthesized by the liver (Pennacchio et al., 2001; van der Vliet et al., 2001; Baroukh et al., 2004; Rensen et al., 2005). After being secreted from the liver, apoA5 is distributed among lipoprotein particles in circulation, wherein this protein promotes triglyceride lipolysis and clearance, resulting in the decrease of plasma triglyceride levels (Schaap et al., 2004; Talmud, 2007; Chen et al., 2021). Studies in human and mice have revealed that apoA5 deficiency are associated with severe hypertriglyceridemia (Priore Oliva et al., 2005; Nelbach et al., 2008). Indeed, apoA5 has been documented as a target of several triglyceride-lowering drugs (Huang et al., 2009; Brautbar et al., 2011), and we previously demonstrated that apoA5 is implicated in the amelioration of hypertriglyceridemia in mice by metformin (Li et al., 2016), a drug for olanzapine-induced glucose and lipid disorder (Wu et al., 2008; Cernea et al., 2020). Together, these data suggested the implication of apoA5 in olanzapine-induced hypertriglyceridemia, which was to be determined in this study.

## 2 Materials and methods

### 2.1 Human studies

#### 2.1.1 Participants

The clinical trial (ClinicalTrials.gov NCT03451734) was conducted at The Second Xiangya Hospital Central South University, China, from January 2018 to June 2019. Schizophrenia was diagnosed according to the criteria established by the Diagnostic and Statistical Manual of Mental Disorders-Fifth Edition (DSM-5) (Battle, 2013). Patients with schizophrenia, between 18 and 60 years of age, experiencing their first psychotic episodes were included in this study.

The exclusion criteria were as follows: 1) current or prior DSM-IV diagnosis of other psychiatric disorders defined in the DSM-5; 2) pregnancy or lactating; 3) history of alcohol, cigarette, or substance use; 4) known medical conditions that might affect metabolism; and 5) history of diabetes, hypertension, cardiovascular diseases, endocrine diseases, lipid disorders, and/or serious chronic diseases, such as heart failure and liver or kidney dysfunction.

This study was performed in accordance with the 1964 Declaration of Helsinki and its later amendments (General Assembly of the World Medical Association, 2014) and was approved by the Ethics Committee of The Second Xiangya Hospital, Central South University. After a complete description of the study to the participants, written informed consents were obtained from them prior to their participation.

#### 2.1.2 Intervention

The participants underwent an 8-week treatment program. The intervention was the intake of olanzapine (15–20 mg/day) monotherapy, administered daily at 8:00 p.m. The initial dose of olanzapine was 5 mg/day, which was then titrated to 15 mg/day to 20 mg/day during the first week.

#### 2.1.3 Assessment

All participants underwent a clinical evaluation with scheduled follow-ups at weeks 4 and 8. The baseline assessments included demographics, comprehensive medical history, physical examination, anthropometric measurements (weight and height), and positive and negative syndrome scale (PANSS) scores. The baseline laboratory tests included assessments of fasting lipid and glucose levels, liver and renal function, and complete blood count, as well as electrocardiography. At each follow-up visit, all baseline clinical evaluations, including physical examination, anthropometric measurements, and laboratory tests, were repeated. The Treatment Emergent Symptom Scale (TESS) (Guy, 1976) was used to record adverse events throughout the clinical trial. PANSS scores were also evaluated at the end of the trial. (Coccurello et al., 2009).

The primary outcomes were changes in fasting plasma triglyceride and apoA5 levels. Plasma apoA5 levels were determined using ELISA kits (NBP2-68250, Novus Biologicals, United States). Blood glucose and lipids, including triglycerides, total cholesterol (TC), LDL-C, and HDL-C, were detected using an automatic biochemical analyzer.

The secondary outcomes were the following: 1) increased levels of other lipids, including TC, LDL-C, and HDL-C; 2) changes in fasting glucose levels and body mass index (BMI); and 3) psychopathological symptoms measured by PANSS scoring. BMI was calculated as the weight in kilograms divided by the square of the height in meters.

#### 2.1.4 Statistical analyses

All continuous variables with normal distributions are expressed as the mean ± standard error of the mean (SEM). Changes in parameters, such as BMI, plasma lipid levels, and apoA5 levels, were defined as the amount of their alterations from the baseline to the endpoint (i.e., 4 and 8 weeks). Continuous variables were analyzed using analysis of variance (ANOVA). Pearson’s analysis was used for the correlation test. A *p*-value was used to indicate statistically significant differences, with a *p* < 0.05 considered significant. The GraphPad Prism statistical software (v.8.0.1; GraphPad Software, La Jolla, CA, United States) and SPSS software (v.25.0; SPSS Inc., Chicago, IL, United States) were used for statistical analyses of all data in this study.

### 2.2 Animal studies

#### 2.2.1 Animals

A total of 21 female C57BL/6J mice (8-weeks old) were purchased from Hunan Stryker Jingda Animal Co., Ltd. (Hunan, China). All animals were housed under standard conditions in individually ventilated cages and an artificial 12/12 h light/dark cycle (lights on: 8:00 a.m.) at 20–25°C and humidity of 45 ± 10%. Mice had *ad libitum* access to food and water. The mice were fed a regular chow diet containing 4.5% fat (0.02% cholesterol) throughout the experimental period. All animal procedures were conducted in the Department of Laboratory Animals in Central South University and performed in accordance with the National Institute Health Guide for the Care and Use of Laboratory Animals. The entire experimental procedure was approved by the Experimental Animal Ethics Committee of The Second Xiangya Hospital, Central South University.

#### 2.2.2 Treatments

Drug administration was initiated after 1 week of acclimatization. The mice were randomly categorized into low-dose olanzapine, high-dose olanzapine, and control groups. Mice in the low-dose olanzapine group (*n* = 7) received 3 mg/kg olanzapine (LY170053, MCE, United States) daily through gavage for 6 weeks, whereas the high-dose group received 6 mg/kg olanzapine for 6 weeks. Olanzapine was dissolved in 10% dimethyl sulfoxide (DMSO), and therefore the control group was maintained on a regular diet with 0.01 ml/g DMSO daily through gavage. The dosages of olanzapine used in this study referenced previous studies (Shertzer et al., 2010; Yoon et al., 2010; Alexander et al., 2011; De Hert et al., 2011; Schmidt et al., 2013).

#### 2.2.3 Body weight, sample collection, and biochemical analyses

The body weights of all animals were assessed weekly during the study. At week 6, animals were fasted for 4 h and then anesthetized with pentobarbital according to AVMA Guidelines for the Euthanasia of Animals: 2020 Edition (Leary et al., 2020)*.* Liver samples were collected, frozen in liquid nitrogen immediately, and stored at -80°C for subsequent analyses. Blood samples were collected from the hearts and placed in ethylene diamine tetraacetic acid-coated tubes. Blood samples were then centrifuged at 1,000 g and 4°C for 15 min to isolate the plasma. The plasma levels of glucose, triglycerides, and TC were measured using a Spotchem EZ SP 4430 (Arkray, Inc., Kyoto, Japan). Plasma apoA5 levels were determined using ELISA kits (A104763; Chemical Book, China).

#### 2.2.4 Glucose tolerance test

At the end of the study, all animals underwent a GTT. Briefly, mice were fasted for 6 h, with only water provided *ad libitum* from 9 a.m. on the experimental day. Before the test of blood glucose, all the mice were weighted. The required volume of 20% glucose solution (1.5 g of glucose/kg body mass) for IP injection was calculated and recorded as follows: V_GTT_ (μL) = 10 X body weight (g). The initial blood glucose levels of mice were assessed 2 h prior to the GTT. During the GTT, blood glucose levels were obtained at 15, 30, 60, 90, and 120 min after the intraperitoneal dose of glucose. Blood samples were drawn from the tail veins and analyzed using a contour glucometer (Bayer Pharma AG, Leverkusen, Germany).

#### 2.2.5 Histologic examination

Frozen liver samples were cut into 6- to 8-µm-thick sections and subjected to histologic examination. Hematoxylin and eosin (H&E) staining was performed on formalin-fixed, paraffin-embedded sections using a standard protocol as described previously (Simon et al., 2020). Oil red O (ORO) staining was conducted to observe the lipid droplets according to the standard ORO staining protocol (ORO, Merck, Germany). An immunofluorescence (IF) assay for lipid droplets and apoA5 protein was performed on frozen liver sections with boron-dipyromethene (BODIPY 493/503) (Molecular Probes, Thermo Fisher Scientific, United States) and primary antibody against apoA5 (apoA5, sc-373950, Santa Cruz Biotechnology, Dallas, TX, United States). The apoA5 antibody was added and incubated for 2 h at room temperature. Goat anti-mouse IgG antibodies conjugated with Alexa fluor 647 (550047, ZEN-BIOSCIENCE, Chengdu, China) were used as secondary antibodies. Then the sections were incubated with BODIPY 493/503 at a concentration of 10 μg/ml for 45 min for neutral lipid staining. For nuclei, 4′,6-diamidino-2-phenylindole (DAPI) (S2110, Beijing Solarbio Science and Technology Co., China) was used. At least three discontinuous liver sections were evaluated for each mouse.

### 2.3 Cell studies

For this study, two cell lines were selected. The primary mouse hepatocyte was chosen because of its involvement in the metabolism of olanzapine. The hepatoma cell line HepG2 (ATCC HB-8065) (Rockville, MD) was selected because of the intense lipid metabolism of this tissue (Canfrán-Duque et al., 2013).

#### 2.3.1 Culture of cells

Primary mouse hepatocytes were isolated and purified from C57BL/6J mice by using a modified two-step perfusion method described in previous studies (Klaunig et al., 1981a; Klaunig et al., 1981b). Briefly, the hepatic portal vein was cannulated and perfused with Krebs Ringer after anesthetization, which contained collagenase IV, for 10 min. After the first wash, a second wash using Krebs Ringer containing CaCl_2_ and Liberase was performed for 10 min. All solutions were heated to 37°C. Hepatocytes were filtered through a gauze mesh and resuspended in Dulbecco’s modified Eagle’s medium (DMEM) containing 10% fetal bovine serum, 1 U/mL penicillin, and 1 mg/ml streptomycin. Cells were plated in 6-well plates and incubated in a humidified atmosphere containing 5% CO_2_ at 37°C. The human hepatoma cell line HepG2 was also cultured in DMEM in an atmosphere containing 5% CO_2_ at 37°C and was passaged every 3 days.

#### 2.3.2 Treatments to HepG2 cells and primary mouse hepatocytes

Both the HepG2 cells and primary mouse hepatocytes were divided into 4 groups: olanzapine 25 µmol/L group, olanzapine 50 µmol/L group, olanzapine 100 µmol/L group, and the control group as described previously (Yang et al., 2007). In the olanzapine groups, the drug was added to the medium for 24 h. Olanzapine was dissolved in DMSO (D2650, Sigma-Aldrich, United States). Approximately 0.1% DMSO was added to the culture medium of control cells.

#### 2.3.3 Measurements of triglyceride levels of the medium

All the cultured medium stated in 2.3.2. was collected. The triglyceride levels of the medium were determined subsequently in the Clinical Laboratory of the second Xiangya hospital using Spotchem EZ SP 4430 (Arkray, Inc., Kyoto, Japan). The corresponding treated hepatocytes were collected, and the protein levels were measured by BCA protein assay kit (CW0014S, CWBIO, China) for the normalization of triglyceride levels in the medium.

#### 2.3.4 Isolation of lipid droplets

To probe the relationship between apoA5 and lipid droplets in hepatocytes, lipid droplets were isolated by sucrose gradient centrifugation as described previously (Brasaemle and Wolins, 2016). The primary mouse hepatocytes and HepG2 cells were cultured with DMEM containing 0.1% DMSO or 100 µmol/L olanzapine. After 24 h, cells were homogenized using the Potter-Elvehjem tissue homogenizer, centrifuged (10 min at 1,000 × g, 4°C), and then were collected into a separate 15-ml tube. Lipid droplets were isolated by adding 1/3 volume of ice-cold HLM-containing 60% sucrose. By using an Autodensiflow gradient fractionator (Labconco) to rinse residual lipid droplets, four 1 ml fractions of lipid droplets were collected from the top of the gradient.

#### 2.3.5 Western blotting analysis

The effects of olanzapine administration on apoA5 protein levels in mice and hepatocytes were determined by Western blotting. The cultured primary mouse hepatocytes and HepG2 cells were washed thrice with ice-cold phosphate-buffered saline (PBS), and radioimmunoprecipitation assay (RIPA) lysis buffer (P0013D, Beyotime Biotechnology, China) containing 1% 0.5 mm phenylmethanesulfonyl fluoride (PMSF) (v/v) was added, after which the cells were incubated at 4°C for 30 min. The cells were then collected and centrifuged at 13,000 g for 15 min at 4°C after being mixed with a pipette. The liver tissues were homogenized in the RIPA lysis buffer (P0013B, Beyotime Biotechnology, China) containing 1% 0.5 mm PMSF (v/v) at 100 mg/ml. Liver tissues were then incubated for 30 min, and the homogenate was centrifuged at 13,000 rpm for 15 min at 4°C, after which the supernatant was saved as a protein extract. Protein levels were quantified using a BCA protein assay kit (CW0014S, CWBIO, China). Protein extracts were mixed with loading buffer and PBS, followed by incubation at 95°C for 5 min to allow degeneration. Protein extracts were then separated by SDS-PAGE and transferred to a polyvinylidene difluoride membrane. To visualize protein samples and to confirm the complete and equivalent transfer of protein samples extracted from the medium, 0.1% Ponceau S (CW0057, CWBIO, China) was applied. After blocking with 5% skimmed milk in Tris-buffered saline with 0.1% Tween-20 (TBST) for 2 h at room temperature, the membranes were incubated overnight at 4°C with primary antibodies (apoA5, #3335, Cell Signaling Technology, United States; apoA5, sc-373950, Santa Cruz Biotechnology, United States; tubulin, 66031-1-Ig, Proteintech, United States). Samples were then incubated with secondary goat anti-mouse and anti-rabbit antibodies (Anti-Mouse, SA00001-1, Proteintech, United States; Anti-Rabbit, SA00001-2, Proteintech, United States). The bound complexes were detected using Pierce ECL Western blotting substrate (32209, Thermo Fisher Scientific, United States) and quantified using a ChemiDoc XRS + system (Bio-Rad, United States).

#### 2.3.6 Real-time quantitative PCR

To further explore the effects of olanzapine on hepatic apoA5 metabolism, *ApoA5/APOA5* mRNA level was measured by qPCR. Total RNA was extracted from mouse liver tissues, primary mouse hepatocytes, and HepG2 cells using an RNA extraction kit (K0731, Thermo Fisher Scientific, United States) according to manufacturer instructions. First-strand cDNA was synthesized using the Revert Aid first-strand cDNA synthesis kit (K1622, Thermo Fisher Scientific, United States). qPCR was performed using SYBR Green select master mix (172-5,121, Bio-Rad, United States), with *glyceraldehyde 3-phosphate dehydrogenase* (*GAPDH*) used as an endogenous control. Primers were synthesized by Tsingke Biological Technology (Beijing, China). Primer sequences are shown in Table 1.

**TABLE 1:** Oligonucleotide sequences of primers for APOA5/ApoA5 and GAPDH.

Gene	Source	Category	Sequences
*APOA5*	Human	Primers	Forward: 5'-GCC AGC GAC TTC AGG CTT T-3'
			Reverse: 5'-AGC TTG CTC AGA ACC TTG CC-3'
*ApoA5*	Mouse	Primers	Forward: 5'-TCC TCG CAG TGT TCG CAA G-3'
			Reverse: 5'-GAA GCT GCC TTT CAG GTT CTC-3'
*GAPDH*	Human	Primers	Forward: 5'-TGT GGG CAT CAA TGG ATT TGG-3'
			Reverse: 5'- ACA CCA TGT ATT CCG GGT CAA T-3'
*Gapdh*	Mouse	Primers	Forward: 5'-TGG CCT TCC GTG TTC CTA C-3'
			Reverse: 5'-GAG TTG CTG TTG AAG TCG CA-3'

### 2.4 Statistical analyses

All statistical analyses were performed using GraphPad Prism software (GraphPad Software) and statistical package IBM SPSS Statistics software (SPSS). Data were expressed as the mean ± SEM. Parametric analyses were conducted using Dunnett’s t test, one-way ANOVA, simple linear regression, Pearson Correlation coefficient. Non-parametric analyses were performed using Wilcoxon rank sum test, Kruskal-Wallis H test, and Spearman’s rank correlation coefficient. When the repeated measured data were normally distributed, the assumptions of repeated measures ANOVA, also called sphericity, would be conducted. Once the assumption accepted, the data would be analyzed by One-way Repeated Measures ANOVA. Otherwise, Geisser-Greenhouse correction would be used to correct for violations of the assumption. Multiple stepwise regression analysis was conducted to screen and eliminate the variables causing multicollinearity. Differences were considered statistically significant at a *p* < 0.05.

## 3 Results

### 3.1 Short-term use of olanzapine increases plasma triglyceride levels and reduces plasma apoA5 levels in schizophrenia patients

**FIGURE 1:**
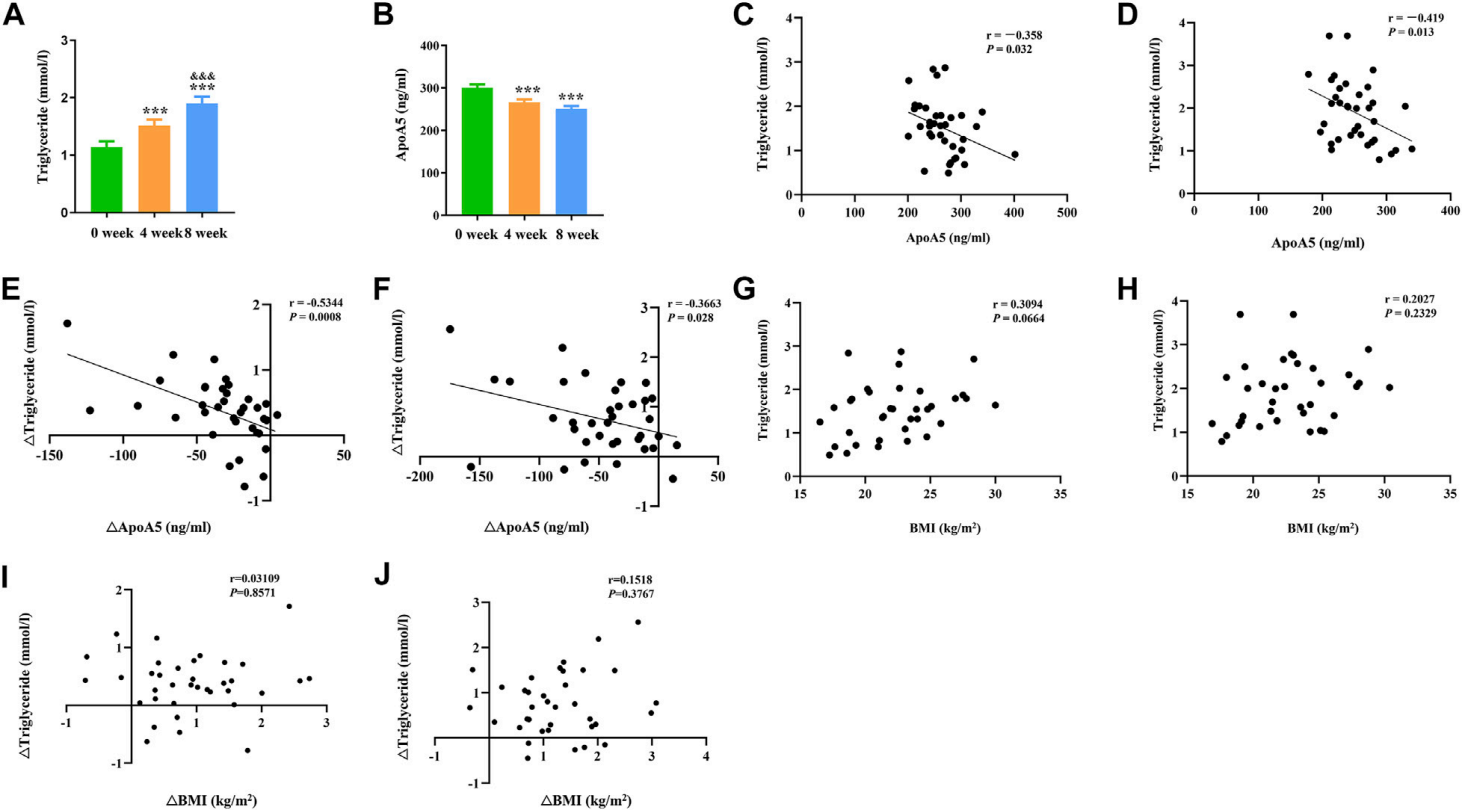
Reduction of plasma apoA5 levels contributed to olanzapine-induced hypertriglyceridemia in schizophrenia patients. **(A)** Plasma triglyceride and **(B)** apoA5 levels in schizophrenia patients. Plasma apoA5 levels correlated with alterations in plasma triglyceride levels after 4-week **(C)** and 8-week **(D)** treatment of olanzapine. Changes in plasma apoA5 levels correlated with alterations in plasma TG levels at week 4 **(E)** and week 8 **(F)**. Results of correlation analyses between BMI and plasma triglyceride levels at week 4 **(G)** and week 8 **(H)**. Results of correlation analyses between changes in BMI and changes in plasma triglyceride levels at week 4 **(I)** and week 8 **(J)**. Results are shown as the mean ± SEM. BMI: body max index; *p < 0.05 vs. control, **p < 0.01 vs. control, ^&^ *p* < 0.05 vs. 4-week olanzapine treatment.

In total, 36 drug-naïve adult patients with schizophrenia (male n = 18, 50.00%) in their first psychotic episode were enrolled in this study. All participants completed the 8-week study. As shown in Figure 1 and Table 2, after short-term (8-week) olanzapine treatment, the patients presented considerably elevated plasma triglyceride levels and reduced plasma apoA5 levels during the follow-up. Notably, the plasma triglyceride levels of these subjects increased as early as week 4. At week 8, 52.78% (19/36) of the patients had hypertriglyceridemia (≥1.7 mmol/L). Over the 8-week study period, the mean triglyceride levels increased by 0.76 mmol/L (Figure 1A), whereas the mean apoA5 levels decreased by 48.87 ng/ml (Figure 1B). There were no gender differences in plasma triglyceride levels or apoA5 levels at week 4 or week 8 (Supplementary Figure S1). Correlation analyses showed that plasma apoA5 levels were negatively correlated with plasma triglyceride levels at week 4 (r = −0.358, *p* = 0.032, Figure 1C) and week 8 (r = −0.419, *p* = 0.013, Figure 1D) after olanzapine treatment. Notably, the reduction of plasma apoA5 levels were also negatively correlated with elevation of plasma triglyceride levels (r = −0.5344, *p* = 0.0008, week 4, Figure 1E; r = −0.3663, *p* = 0.028, week 8, Figure 1F). However, plasma triglyceride levels did not correlate with BMI (r = 0.3094, *p* = 0.0664, week 4, Figure 1G; r = 0.2027, *p* = 0.2359, week 8, Figure 1H), and the changes in plasma triglyceride levels did not correlate with that in BMI (r = 0.03109, *p* = 0.8571, week 4, Figure 1I; r = 0.1518, *p* = 0.3767, week 8, Figure 1J). Stepwise multiple regression analysis of the independent correlation for plasma triglycerides showed that plasma apoA5 levels were independently associated with plasma triglyceride levels at week 4 (*p* = 0.001) and week 8 (*p* = 0.028) after olanzapine treatment (Table 3).

**TABLE 2:** Clinical and biochemical characteristics of schizophrenia patients receiving olanzapine therapy.

	Baseline	4 weeks	8 weeks
Female, n (%)	18 (50)	18 (50)	18 (50)
Weight (kg)	59.72 ± 10.59	62.33 ± 11.09	65.18 ± 11.23
BMI (kg/m2)	21.39 ± 3.33	22.30 ± 3.36	23.32 ± 3.33*
Systolic blood pressure (mmHg)	117.61 ± 9.08	117.25 ± 9.09	116.14 ± 8.69
Diastolic blood pressure (mmHg)	71.11 ± 6.79	71.50 ± 4.79	70.78 ± 7.14
Fasting plasma glucose (mmol/L)	4.58 ± 0.69	4.56 ± 0.84	4.990 ± 0.74
Triglycerides (mmol/L)	1.14 ± 0.60	1.51 ± 0.62*	1.90 ± 0.74*
Total cholesterol (mmol/L)	4.05 ± 0.80	4.29 ± 0.71	4.49 ± 0.74*
HDL-C (mmol/L)	1.26 ± 0.34	1.29 ± 0.26	1.27 ± 0.29
LDL-C (mmol/L)	2.40 ± 0.56	2.54 ± 0.53	2.71 ± 0.53*
apoA5 (ng/ml)	299.75 ± 48.58	265.94 ± 40.51*	250.88 ± 37.35*

*p < 0.05 vs. baseline.

BMI, body max index; HDL-C, high-density lipoprotein cholesterol; LDL-C, low-density lipoprotein cholesterol.

**TABLE 3:** Stepwise multiple regression analysis detecting independent contributors to plasma triglyceride levels in schizophrenia patients.

Factor	*β* ^*#*^	*p*	*β* ^*^	*p*
Weight	−0.039	0.796	0.068	0.697
BMI	−0.052	0.731	−0.014	0.933
Fasting plasma glucose	−0.272	0.060	0.062	0.707
Total cholesterol	−0.215	0.158	−0.068	0.675
HDL-C	−0.241	0.110	−0.151	0.354
LDL-C	−0.291	0.062	−0.136	0.422
apoA5	−0.534	0.001	−0.366	0.028

*4 weeks vs. baseline.

^#^8 weeks vs. baseline.

β, standardized regression coefficients. a dependent variable: plasma triglyceride levels.

BMI, body max index; HDL-C, high-density lipoprotein cholesterol; LDL-C, low-density lipoprotein cholesterol.

### 3.2 Changes in body weight, plasma cholesterol, and glucose levels in schizophrenia patients

After 8 weeks of olanzapine treatment, the mean BMI of these patients increased by 1.93 kg/m^2^ but remained within the normal range (18.5–24 kg/m^2^) (Supplementary Figure S2A). Plasma TC and LDL-C levels markedly increased (Supplementary Figure S2B,C), whereas no substantial changes in plasma HDL-C and fasting glucose were observed in these patients after olanzapine treatment (Supplementary Figure S2D,E). Meanwhile, no significant correlation between the BMI and any other metabolic indicators, including circulating cholesterol (total cholesterol (TC), LDL-C, and HDL-C), and glucose after 4- (Supplementary Figure S3A–D) or 8-week (Supplementary Figure S3E–H) of olanzapine treatment. And further analyses showed that there was no significant correlation between the BMI and lipids or fasting blood glucose in schizophrenia patients after 4- (Supplementary Figure S4A–D) or 8-week (Supplementary Figure S4E–H), either.

### 3.3 Olanzapine treatment affects body weight, glucose tolerance, plasma lipid and apoA5 levels in mice

**FIGURE 2:**
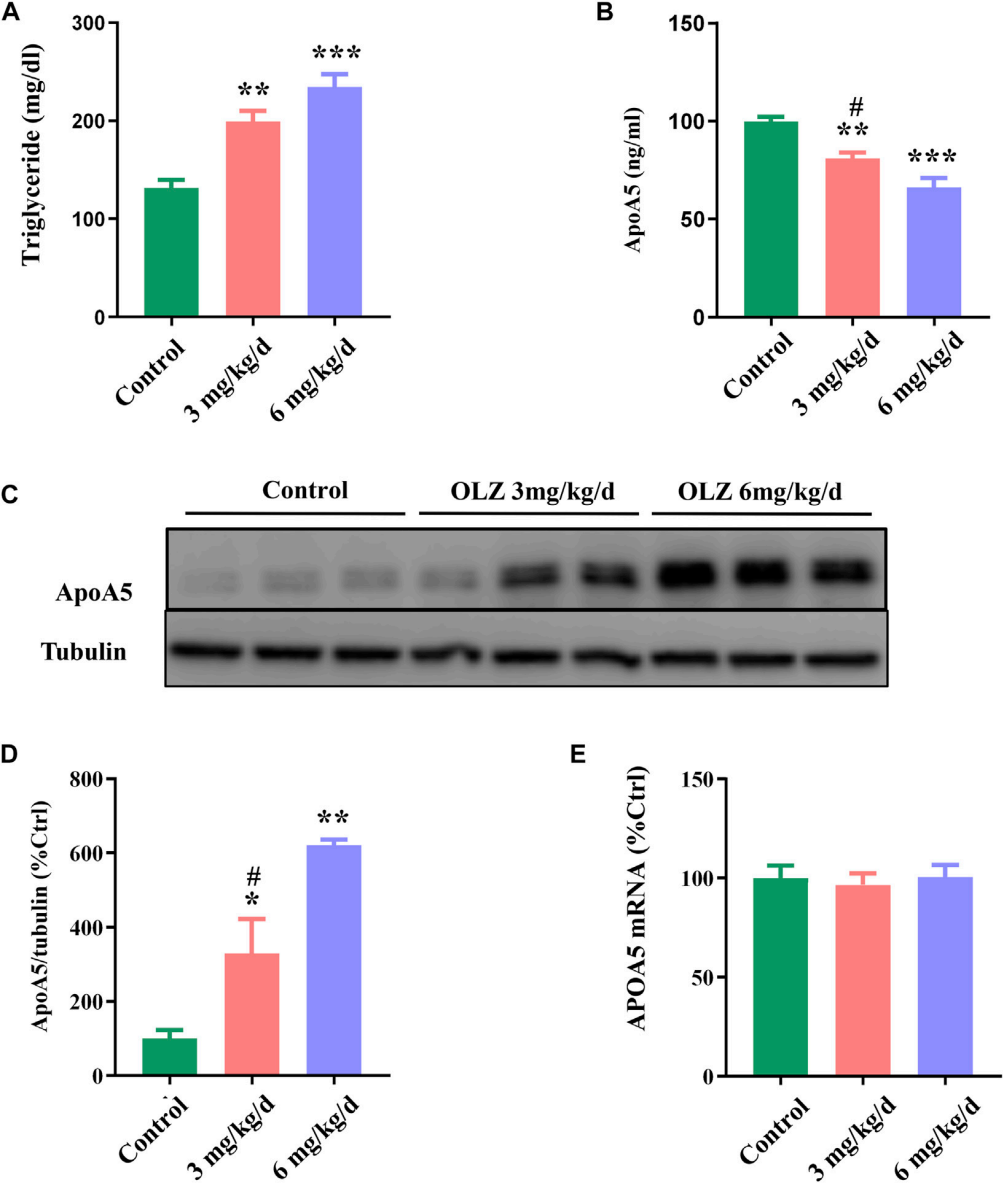
The effects of olanzapine (OLZ) on plasma triglyceride and apoA5 levels in mice. **(A)** Plasma triglycerides and **(B)** apoA5 levels in olanzapine-treated mice. Hepatic apoA5 protein **(C,D)** and *ApoA5* mRNA levels **(E)** in olanzapine-treated mice. Data represent the mean ± SEM. *p < 0.05 vs. control, **p < 0.01 vs. control, ^#^ *p* < 0.05 vs. OLZ 6 mg/kg group.

Considering the relevant changes in schizophrenia patients, we have evaluated the relative parameters and apoA5 expression in a mouse model fed with olanzapine. In this study, we found that olanzapine slightly induced weight gain, but there was no statistic difference between the 3 groups (Supplementary Figure S5A,B), and olanzapine intervention impaired glucose tolerance in mice in a dose-dependent manner (Supplementary Figure S5C,D). Consistent with observations in humans, increased plasma triglyceride levels were observed in mice in two olanzapine groups (low-dose olanzapine group, 199.24 ± 28.88 mg/dl; high-dose olanzapine group, 234.26 ± 35.28 mg/dl) (Figure 2A) relative to the control group (131.35 ± 22.43 mg/dl). Compared with the low-dose olanzapine group, plasma triglyceride levels were greatly increased in the high-dose olanzapine group (*p* < 0.05). Moreover, the results demonstrated that olanzapine administration dose-dependently decreased plasma apoA5 levels in mice (low-dose olanzapine group, 80.90 ± 8.22 ng/ml; high-dose group olanzapine group, 66.14 ± 13.19 ng/ml; and control group, 99.76 ± 6.54 ng/ml; *p* < 0.05) (Figure 2B).

### 3.4 Olanzapine increases hepatic apoA5 protein levels without changes in the *ApoA5* mRNA levels in mice

To investigate the role of apoA5 in olanzapine-induced hypertriglyceridemia, we measured apoA5 expression at both the protein and the mRNA levels in mouse livers. Hepatic apoA5 protein levels were markedly increased in mice after 8 weeks of olanzapine treatment in a dose-dependent manner (*p* < 0.05) (Figures 2C,D). Interestingly, there were no significant changes in hepatic *ApoA5* mRNA level among the three groups (*p* > 0.05) (Figure 2E).

### 3.5 Olanzapine treatment increases hepatic apoA5 protein *in vitro* without changing hepatic *APOA5/ApoA5* mRNA

**FIGURE 3:**
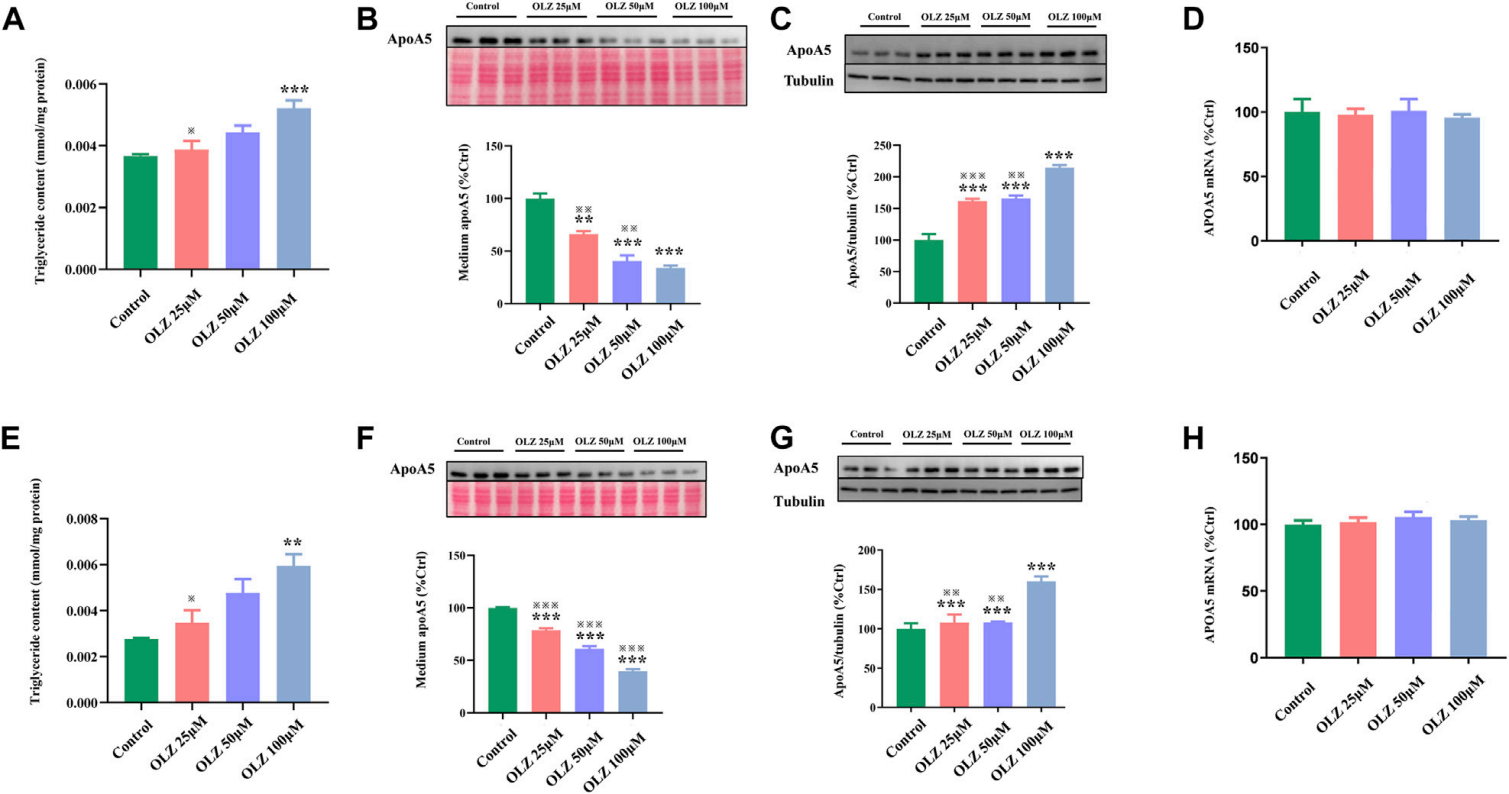
The effects of olanzapine (OLZ) on medium triglyceride levels, apoA5 protein and *APOA5/apoA5* mRNA expression in human and mouse hepatocytes *in vitro*. Triglyceride levels of medium in human hepatocytes **(A)**. Intracellular apoA5 protein levels **(B)**, apoA5 protein levels in the medium **(C)** (70 μg protein was loaded per lane and loading was confirmed by Ponceau-staining**)** and *APOA5* mRNA **(D)** in HepG2 cells. Triglyceride levels of medium in primary mouse hepatocytes **(E)**. Intracellular apoA5 protein levels **(F)**, apoA5 protein levels in the medium **(G)** (100 μg protein was loaded per lane and loading was confirmed by Ponceau-staining**)** and *ApoA5* mRNA **(H)** in primary mouse hepatocytes. Data represent the mean ± SEM. ^*^ *p* < 0.05 vs. control, ^**^ *p* < 0.01 vs. control, ^※※^ *p* < 0.01 vs. olanzapine (OLZ) 100 µM group, ^※※※^ *p* < 0.001 vs. olanzapine (OLZ) 100 µM group.

To further investigate the findings of triglyceride and apoA5 *in vivo*, we detected the effects of olanzapine on triglyceride levels, apoA5 protein levels, and *APOA5/ApoA5* mRNA expression in human and mouse hepatocytes *in vitro*. In line with the findings *in vivo*, olanzapine intervention elevated the medium triglyceride levels in a dose-dependent manner (Figure 3A,E), and decreased apoA5 levels in the medium of human and mouse hepatocytes *in vitro* (Figures 3B,F). Moreover, olanzapine treatment increased the hepatic apoA5 protein levels (Figures 3C,G). Of note, the hepatocellular *APOA5/ApoA5* mRNA expression was not significantly altered after olanzapine intervention (Figures 3D,H).

### 3.6 Elevated hepatocyte apoA5 protein leads to lipid droplet formation and hepatocyte steatosis

**FIGURE 4:**
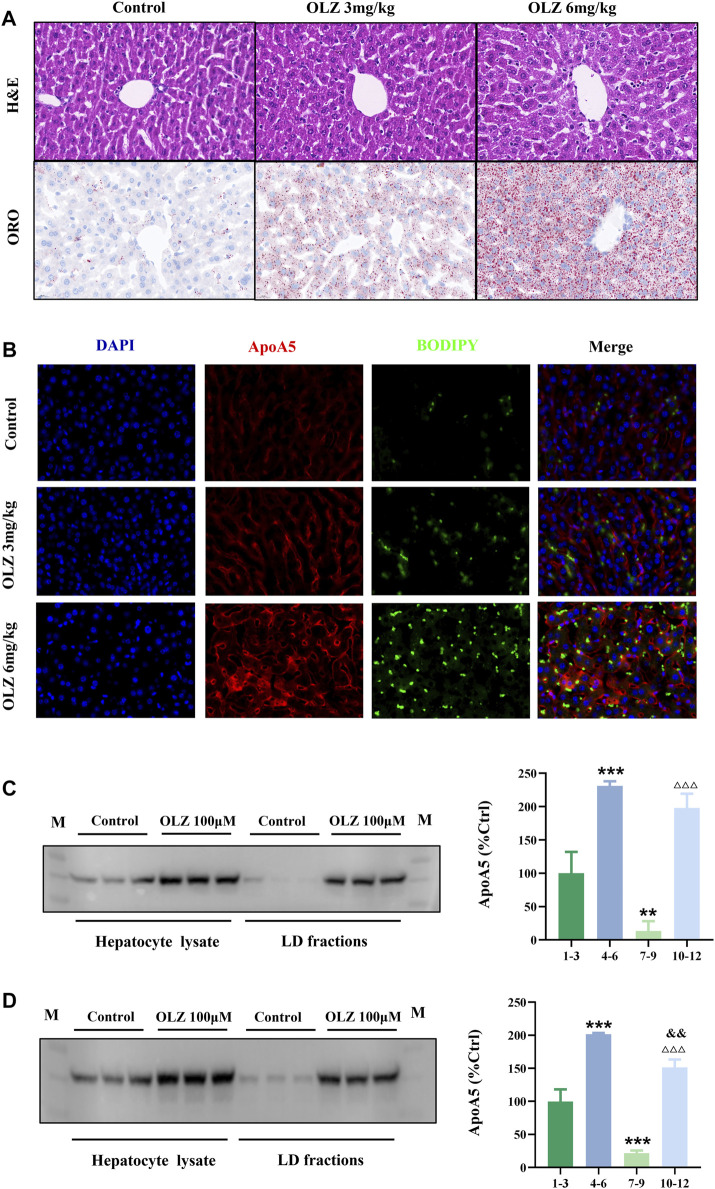
Effects of olanzapine-induced triglyceride accumulation in the liver and on apoA5 subcellular localization alternation. **(A)** H&E and ORO staining and **(B)** double immunofluorescence of mouse liver sections. The liver sections were fixed, permeabilized, and incubated with a mouse monoclonal anti-apoA5 antibody, followed by incubation with Alexa 647-conjugated anti-mouse IgG (red). The nuclei of the corresponding cells were visualized using 4′,6-diamidino-2-phenylindole (DAPI) staining (blue). Lipid droplets were stained with BODIPY 493/503 (green). HepG2 cells **(C)** and primary mouse hepatocytes **(D)** were homogenized and subjected to sucrose gradient centrifugation (Klaunig et al., 1981a). Western blotting results against apoA5 antibody. Lanes 1-3, proteins from the whole cell in the control group; lanes 4-6, proteins from whole cell in the olanzapine 100 µmol/L group; lanes 7-9, proteins from the lipid droplet fractions in the control group; lanes 10-12, proteins from the lipid droplet (LD) fractions in the olanzapine 100 µmol/L group. Data represent the mean ± SEM. LD: lipid droplet. ^**^ *p* < 0.01 vs. lanes 1-3, ^***^ *p* < 0.001 vs. lanes 1-3, ^△△△^ *p* < 0.001 vs. lanes 7-9. ^and&^ *p* < 0.01 vs. lanes 4-6.

We investigated the intrahepatocyte distribution of increased apoA5 protein due to olanzapine treatment. H&E and ORO staining of liver tissues showed that olanzapine markedly promoted the formation of hepatocyte lipid droplets in mice (Figure 4A). Compared with mice in the low-dose olanzapine group, the number of hepatocyte lipid droplets in the high-dose group dramatically increased, and some lipid droplets even fused into larger ones. Additionally, we performed fluorescence double-staining of lipid droplets and apoA5 in these liver sections. As shown in Figure 4B, olanzapine treatment promoted lipid droplet accumulation and induced increase of apoA5 protein in hepatocytes. Notably, after 6-weeks of olanzapine administration, the accumulation of apoA5 was mainly localized in hepatocytes and co-localized with lipid droplets as shown by double immunofluorescence, whereas the apoA5 was mainly localized at the plasma membrane in the control group. Western blotting was performed to confirm that the upregulated apoA5 co-localized with the lipid droplets in hepatocytes (Figures 4C,D). Compared with the apoA5 proteins from the whole cell in the control group, the proteins from the lipid droplet fraction in the control group can barely be seen. In line with the double immunofluorescence results, relative to that of controls, olanzapine treatment resulted in an increase in the apoA5 content in lipid droplets.

## 4 Discussion

For a long time, dyslipidemia after long-term use of olanzapine has mostly been attributed to weight gain associated with olanzapine-induced diet and lifestyle changes (Lieberman et al., 2005; Zhao et al., 2007). However, our study, for the first time, revealed that short-term treatment of olanzapine induced hypertriglyceridemia that was independent of weight gain in schizophrenia patients and mice. Short-term administration of olanzapine (8 weeks) did not lead to obesity in any schizophrenia patients (average BMI: 18.524 kg/m^2^) in this study. Consistently, no significant weight gain was observed in olanzapine-treated mice. The stepwise multiple regression analysis showed no significant correlation between BMI and plasma triglyceride elevation in patients and mice. Further, reduction of plasma apoA5 levels was observed in schizophrenia patients and mice after short-term treatment of olanzapine. More importantly, we showed that olanzapine directly disturbed hepatocyte apoA5 metabolism, resulting in decreased plasma apoA5 levels and increased triglyceride levels. Together, these data suggested that apoA5 plays an important role in olanzapine-induced hypertriglyceridemia.

ApoA5, a member of the exchangeable apolipoprotein family, plays a key role in triglyceride homeostasis (Maher et al., 2011; Nordestgaard and Varbo, 2014). After being specifically synthesized in the liver, apoA5 is secreted into the bloodstream (Pennacchio et al., 2001; van der Vliet et al., 2001; Rensen et al., 2005), where it activates lipoprotein lipase (LPL)-mediated triglyceride hydrolysis for plasma triglyceride reduction (Pennacchio et al., 2001; Merkel et al., 2005; Nilsson et al., 2011; Forte and Ryan, 2015; Chen et al., 2021). The negative regulation of apoA5 on plasma triglyceride levels has been well-documented in human observational studies (Yamada et al., 2007) and genetic engineering animal studies (Pennacchio and Rubin, 2003; Baroukh et al., 2004). They indicated that the triglyceride-lowering efficiency of apoA5, to a large extent, depends on the abundance of circulating apoA5 particles (i.e., plasma apoA5 levels). Given that apoA5 is a liver-specific protein, we initially speculated that olanzapine-induced reduction of plasma apoA5 levels could be a consequence of decreased hepatic apoA5 production, leading to hypertriglyceridemia. Surprisingly, no changes in *APOA5/Apoa5* mRNA expressions were observed in the liver of mice and hepatocytes *in vitro* after olanzapine administration, albeit with a significant increase in hepatic apoA5 protein levels *in vivo* and *in vitro*. Together, our study identified an unexpected paradox of olanzapine on apoA5 metabolism, *i.e.,* olanzapine induced reduction in plasma apoA5 levels, increase in hepatic apoA5 levels, and no change to the expression of hepatic *APOA5* gene. As stated above, apoA5 is a protein specifically synthesized and secreted by the liver (Pennacchio et al., 2001; Rensen et al., 2005; Yamada et al., 2007), and the bloodstream is the major arena wherein apoA5 plays a role in the downregulation of plasma triglyceride levels (Merkel et al., 2005; Rensen et al., 2005; Nilsson et al., 2011; Forte and Ryan, 2015; Chen et al., 2021), which indicated that plasma triglyceride homeostasis depends on the efficiency of the synthesis and secretion of hepatic apoA5. Therefore, our findings indicated that olanzapine-induced reduction of plasma apoA5 levels could result from the retardation of hepatic apoA5 secretion (rather than hepatic apoA5 production/synthesis), which consequently led to hypertriglyceridemia.

ApoA5 is composed of a lipid-surface-seeking C-terminal domain, and an α-helix bundle-forming N-terminal domain. The N-terminal domain plays a role of a functional signal peptide, which guides the apoA5 into the endoplasmic reticulum (ER)-Golgi secretion pathway (Nilsson et al., 2011). The overall hydrophobicity of the apoA5 amino acid sequence, particularly its central and C-terminal regions (Weinberg et al., 2003), may influence protein trafficking in the secretory pathway (Sharma et al., 2012). Under normal conditions, apoA5 is located at the hepatocellular plasma membrane for secretion. Since the short-term use of olanzapine increased apoA5 levels in liver but decreased that in plasma, and our *in vitro* results showed that only a small fraction of newly synthesized apoA5 is secreted from hepatocytes into medium, the evidence suggested that inefficient secretion of apoA5 from the liver into the plasma compartment may, in part, contribute to its low plasma concentration. Indeed, it has been reported that a mutation in the *APOA5* gene (C.16_39del; P. ala6_ala13del) in a breast-fed infant resulted in changes in the signal peptide. The mutated apoA5 was intracellularly missorted to lipid droplets and not secreted, leading to undetectable apoA5 plasma levels, and consequently severe hypertriglyceridemia (Albers et al., 2014). Based on the above, we hypothesized a mechanism for olanzapine-induced hypertriglyceridemia caused by the defects of sorting and secretion of hepatic apoA5, resulting in low plasma apoA5 levels, in which the dysfunctional signal peptide may be involved. Normally, the signal peptide directs the newly synthesized apoA5 into the ER compartment, and then apoA5 is transported to the plasma membrane for secretion. The ER compartment is also the site of lipid droplet genesis (Martin and Parton, 2006; Ploegh, 2007). Due to the dysfunction of the apoA5 signal peptide and its high lipid affinity, apoA5 is missorted as lipid droplets while transiting the ER and binds to nascent lipid droplets, and is subsequently retained on mature cytoplasmic droplets, resulting in secretion inefficiency, and thus reduced plasma apoA5 levels. Therefore, olanzapine treatment disturbs the intracellular trafficking and secretion of apoA5.

This assumption is consistent with the seemingly paradoxical roles of apoA5 in extrahepatic and intrahepatic triglyceride metabolism, as apoA5 reduces plasma triglyceride levels and promotes hepatocyte triglyceride content accumulation (Priore Oliva et al., 2005). In hepatocytes, apoA5 suppresses hydrolysis of triglycerides and facilitates the accumulation of lipid droplets, resulting in the pathogenesis of nonalcoholic fatty liver disease (NAFLD), a disease characterized by excessive triglyceride-rich lipid droplets in hepatocytes (Zheng et al., 2013; Wu et al., 2016). Thus, olanzapine-induced disturbance of intracellular trafficking and secretion of apoA5 potentially contributes to the development of NAFLD in schizophrenia patients, which had been verified in olanzapine-treated mice and supported by other studies (Schaap et al., 2004; Grosskopf et al., 2005; Li et al., 2021). As shown in Figure 4B, in addition to the increased apoA5 and augmented lipid droplets in mice liver sections, it was also observed that apoA5 mainly localized at the plasma membrane in the control group, whereas apoA5 in the olanzapine group co-localized with lipid droplets. Moreover, the determination of apoA5 in lipid droplets of hepatocytes *in vitro* (Figures 4C,D) also suggested that olanzapine treatment dose-dependently increased the apoA5 protein levels in lipid droplets. Notably, there was little apoA5 proteins from the lipid droplet fraction in the control group, showing that normally, apoA5 mainly distributed in the cytoplasm and cell membrane rather than co-localized with lipid droplets. These data further supported the hypothesis that apoA5 is missorted to co-localize with lipid droplets and indicated the translocation of apoA5 to lipid droplets may directly compete with the exocytic trafficking of apoA5 after olanzapine intervention, ultimately resulting in a disturbed distribution of apoA5 in the liver and plasma.

In addition, there had been evidence to support the positive correlation between apoA5 expression and the formation of cytoplasmic lipid droplets, while intracellular triglycerides accumulated at the expense of triglyceride-rich lipoprotein (TRL) secretion (Blade et al., 2011; Gao et al., 2012). Indeed, apoA5 is the only hepatocyte-derived protein known to exist on lipid droplets as well as plasma lipoproteins, and no other hepatocyte lipid droplet associated protein is synthesized with a signal peptide that targets it to the ER lumen (Gao et al., 2012). The elevated intracellular apoA5 functions as a rheostat that directs triglycerides away from secretion towards cytosolic lipid droplet assembly, reducing intrahepatic triglyceride secretion. Because of the high lipid affinity of apoA5, accumulated intrahepatic triglycerides can further antagonize its secretion (Blade et al., 2011). Based on the facts stated above, we could speculate that olanzapine administration increases the content of apoA5 in hepatocytes, leading to the accumulation of triglycerides, which in turn further inhibits apoA5 secretion, and increases intracellular apoA5. Our fluorescence double-staining results confirmed this phenomenon. Therefore, the synthesis and accumulation of triglycerides in the liver could lead to dynamic competition between apoA5 secretion and apoA5 association with lipid droplets, creating a vicious cycle. Meanwhile, reduced apoA5 secretion resulted in decreased plasma apoA5 levels and suppression of hydrolysis for plasma triglyceride, ultimately leading to hypertriglyceridemia. Concomitantly, olanzapine-induced hepatic lipid accumulation is associated with activation of sterol regulatory element-binding protein (Schaap et al., 2004), a transcription factor involved in hepatic *APOA5* expression (Feng et al., 2015). These data suggested a potential link between hepatic apoA5 retention and lipid accumulation due to olanzapine, which can promote NAFLD pathogenesis in schizophrenia patients.

In summary, our *in vivo* and *in vitro* data established that mRNA levels of apoA5 remained unaffected, short-term use of olanzapine significantly inhibited apoA5 secretion and redirected the trafficking of apoA5 to the surface of cytosolic lipid droplets, and reduced plasma apoA5 levels led to hypertriglyceridemia. Namely, olanzapine intervention did not directly affect the transcription of apoA5. It may impair the apoA5 sorting and secretion process from hepatocytes to plasma at the post-translation level, in which the dysfunctional signal peptide of apoA5 may be involved (Romenskaia et al., 2019).

**FIGURE 5:**
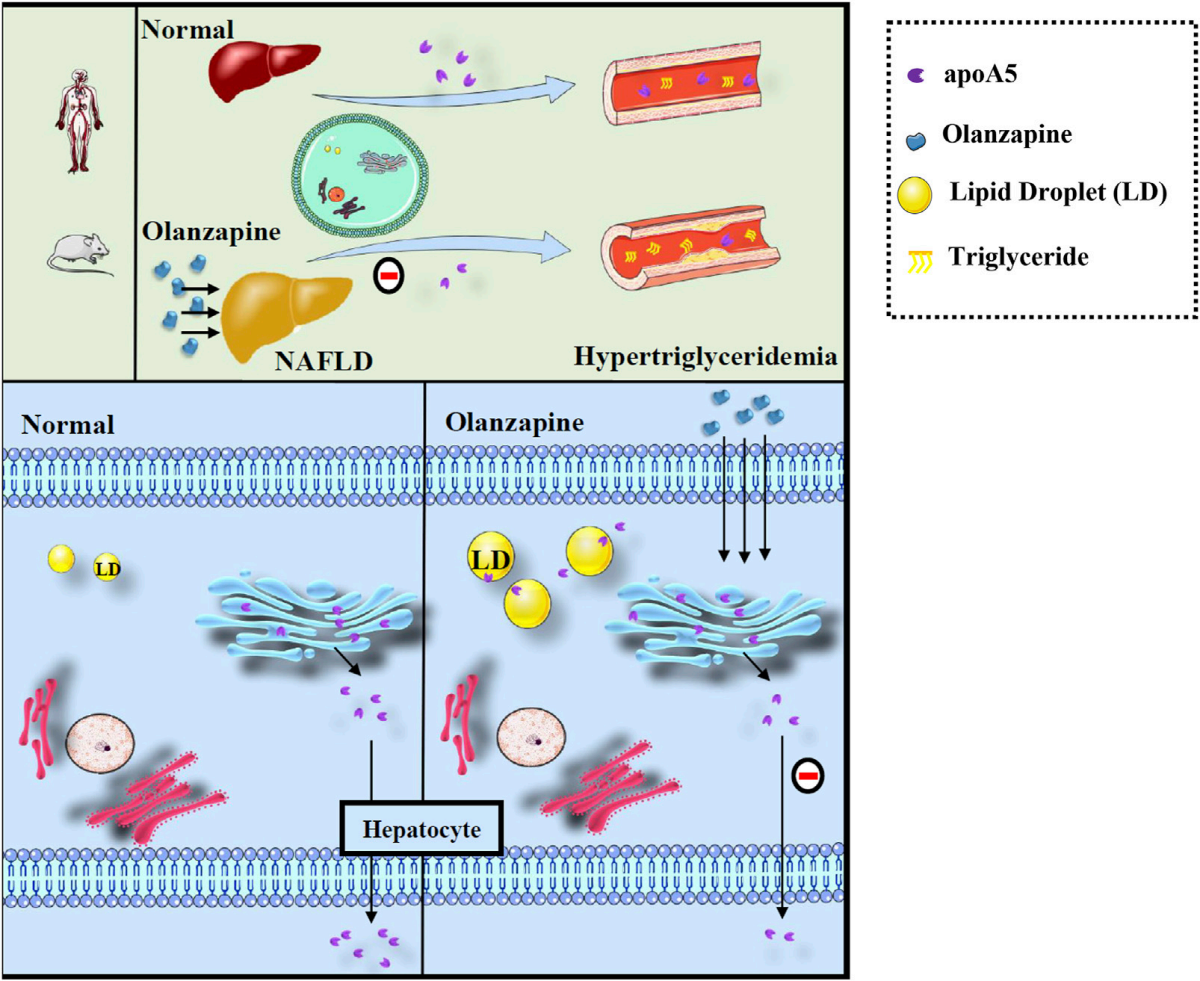
Defects of sorting and secretion of hepatic apoA5 by olanzapine leads to hypertriglyceridemia and lipid droplets accumulation in the liver. ApoA5 is synthesized in the endoplasmic reticulum of hepatocytes and then transported to the Golgi body, which releases it, as needed. Olanzapine intervention does not affect the processes of apoA5 transcription and translation but disturbs hepatic sorting and secretion of apoA5 into the blood, and the reduction of circulating apoA5 results in hypertriglyceridemia. Moreover, apoA5 accumulation in the liver promotes the formation of lipid droplets (LD), inhibits lipid-droplet hydrolysis, and promotes triglyceride accumulation in hepatocytes, potentially increasing the risk of NAFLD.

Here, we proposed a hypothesis for the role of apoA5 in hypertriglyceridemia induced by short-term use of olanzapine (Figure 5). Physiologically, apoA5 is synthesized in the cytosol and co-translationally translocated to the ER and the Golgi body in hepatocytes and secreted into the blood to reduce circulating triglyceride levels. However, olanzapine treatment led to defective hepatic apoA5 sorting and secretion, thereby reducing plasma apoA5 levels and resulting in hypertriglyceridemia. Concomitantly, inhibition of hepatic apoA5 secretion led to hepatocyte apoA5 retention, which facilitates the biogenesis of triglyceride-rich lipid droplets and consequently NAFLD development. We speculated that short-term olanzapine intervention helped apoA5 re-direct from hepatic secretion and become associated with cytosolic lipid droplet formation, which ultimately affects the whole-body triglyceride homeostasis.

Our current work on short-term use of olanzapine *in vitro* and *in vivo* had determined a novel perspective to explain lipid metabolism disorders in clinical schizophrenia patients treated with olanzapine and provided a promising therapeutic target for the future.

## 5 Limitation

Both the clinical and animal experiments were short-term studies, and the changes of plasma apoA5 and triglycerides should be observed over a longer intervention period in further research. What’s more, *APOA5/ApoA5* knockdown tests can also be carried out *in vivo* and *in vitro* to observe the changes of plasma and medium triglycerides in olanzapine intervention. In addition, the mechanism of apoA5 secretion disorder was not further explored in this study.

## Data Availability

The original contributions presented in the study are included in the article/Supplementary Material, further inquiries can be directed to the corresponding author.
