# Supplementary material for: Alterations in sorting and secretion of hepatic apoA5 induce hypertriglyceridemia due to short-term use of olanzapine
Source: Front Pharmacol. 2022 Aug 12;13:935362. doi: 10.3389/fphar.2022.935362 (PMC9411997; doi:10.3389/fphar.2022.935362)
Supplement: Supplementary file 1 [file DataSheet1.pdf]

## Supplementary Information:

Supplementary Figure 1 describes the comparisons between gender in plasma triglycerides and apoA5 levels after olanzapine treatment. Supplementary Figure 2 describes the correlations between BMI and lipids or fasting blood glucose in schizophrenia patients after olanzapine treatment. Supplementary Figure 3 describes the correlations between changes in BMI and changes in lipids or fasting blood glucose in schizophrenia patients after olanzapine treatment. Supplementary Figure 4 describes the effects of olanzapine treatment on plasma lipid and glucose levels in schizophrenia patients. Supplementary Figure 5 shows the effects of olanzapine on body weight, glucose tolerance and blood lipids in mice.

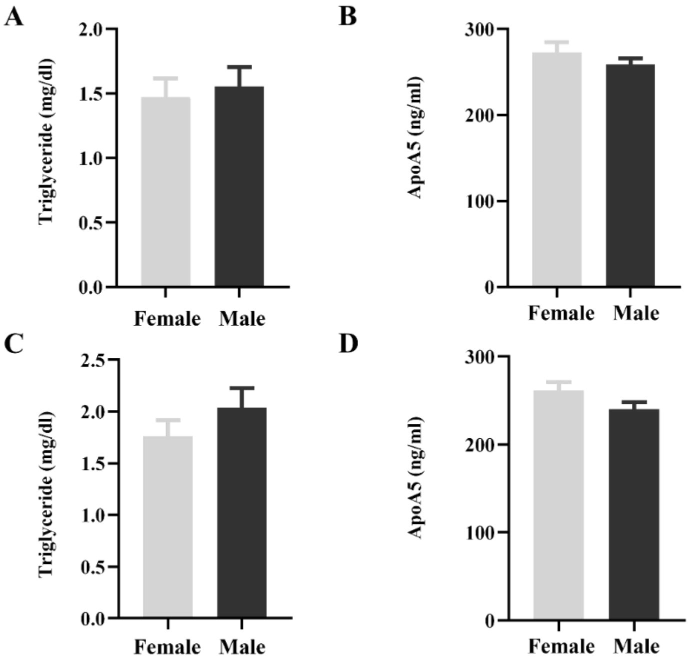


***Supplementary Figure 1. No difference between plasma triglycerides and apoA5 levels in female and male patients after olanzapine treatment. (A)*** *Comparison of plasma triglycerides levels in female and male patients after 4-week olanzapine treatment.* ***(B)*** *Comparison of plasma apoA5 levels in female and male patients after 4-week olanzapine treatment.* ***(C)*** *Comparison of plasma triglycerides levels in female and male patients after 8-week olanzapine treatment.* ***(D)*** *Comparison of plasma apoA5 levels in female and male patients after 8-week olanzapine treatment.*

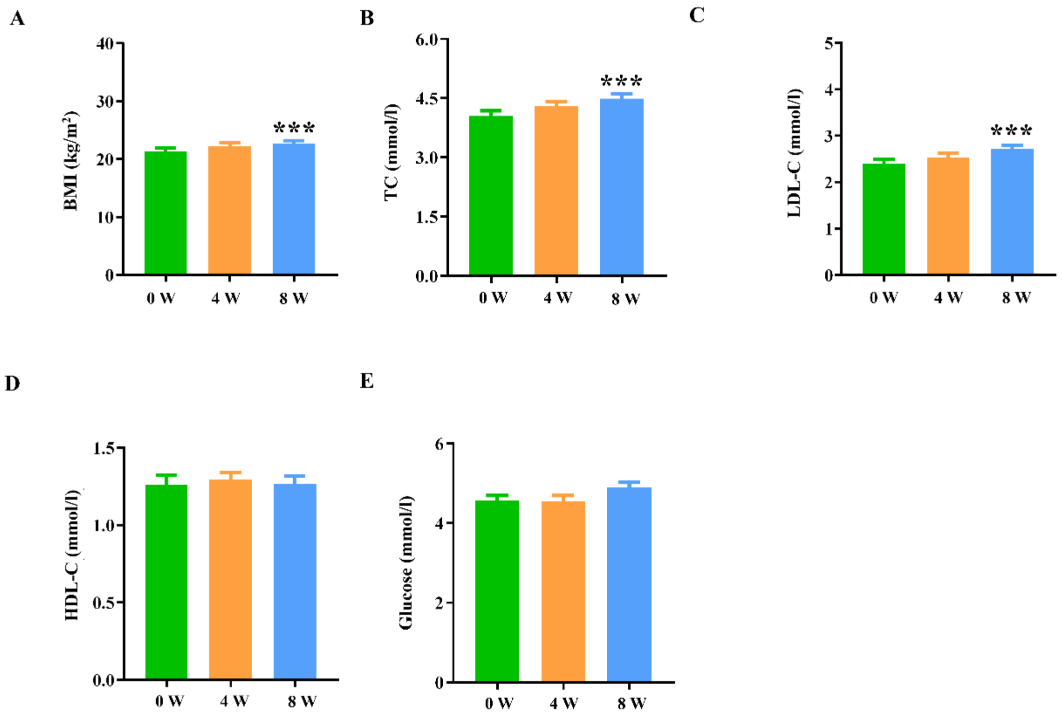


***Supplementary Figure 2. Effects of olanzapine treatment on plasma lipid and glucose levels in schizophrenia patients.*** *(A) BMI. (B) Total cholesterol (TC) levels.* ***(C)*** *High-density lipoprotein cholesterol (HDL-C) levels* ***(D)*** *Low-density lipoprotein cholesterol (LDL-C) levels.* ***(E)*** *Glucose levels. Results are shown as mean ± SEM. BMI: Body mass index. *P < 0.05 vs. control.*

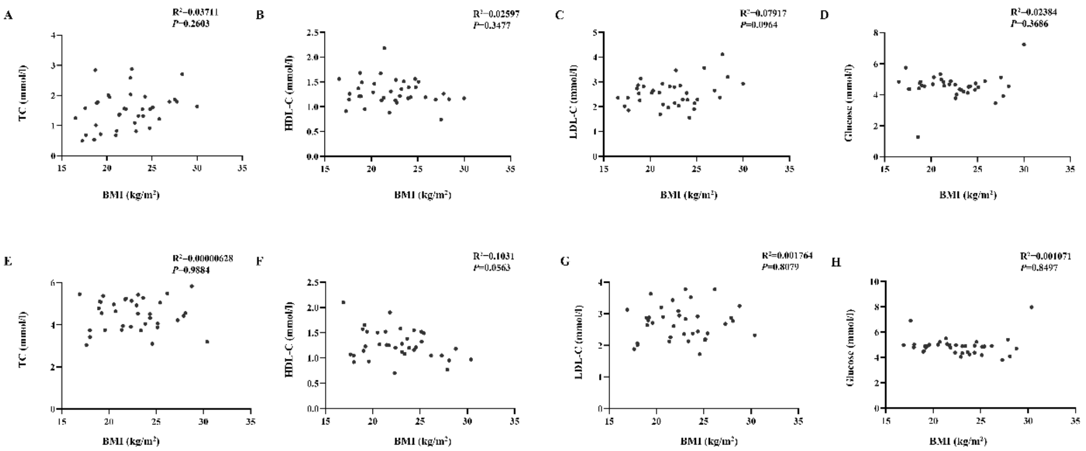


***Supplementary Figure 3. The correlations between BMI and lipids or fasting blood glucose in schizophrenia patients after olanzapine treatment.*** *The correlation between BMI and TC*

***(A)**, HDL-C **(B)**, LDL-C **(C)** and glucose **(D)** after 4-week of olanzapine treatment. The correlation between BMI and TC **(E)**, HDL-C **(F)**, LDL-C **(G)** and glucose **(H)** after 8-week of olanzapine treatment. BMI: body max index; TC: total cholesterol; HDL-C: high-density lipoprotein cholesterol; LDL-C: low-density lipoprotein cholesterol.*

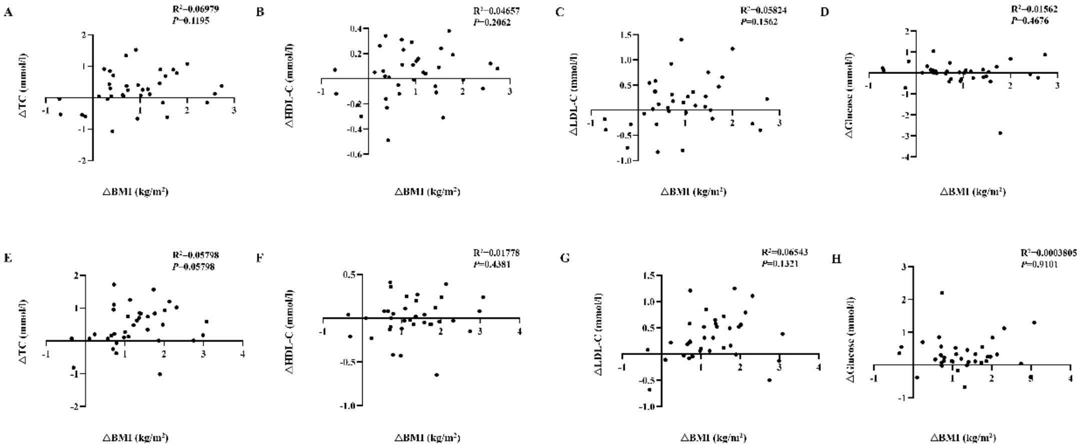


***Supplementary Figure 4. The correlations between changes BMI and changes in lipids or fasting blood glucose in schizophrenia patients after olanzapine treatment.*** *The correlation between changes in BMI and changes in TC **(A)**, HDL-C **(B)**, LDL-C **(C)** and glucose **(D)** after 4-week of olanzapine treatment. The correlation between changes in BMI and changes in TC **(E)**, HDL-C **(F)**, LDL-C **(G)** and glucose **(H)** after 8-week of olanzapine treatment. BMI: body max index; TC: total cholesterol; HDL-C: high-density lipoprotein cholesterol; LDL-C: low-density lipoprotein cholesterol.*

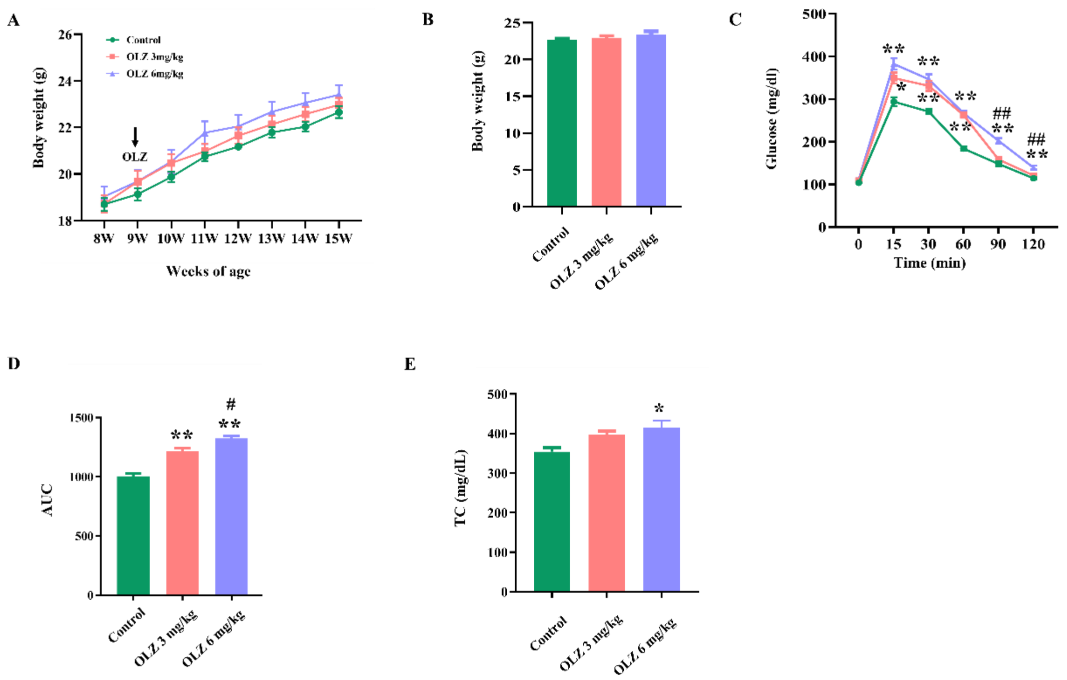


***Supplementary Figure 5. Effect of olanzapine on body weight, glucose tolerance and blood lipids in mice*** *(n = 7 each group).* ***(A)*** *Body weight.* ***(B)*** *Weight gain from baseline.* ***(c)****Glucose tolerance test (GTT).* ***(D)*** *Area under the curve (AUC) of GTT.* ***(E)*** *Plasma total cholesterol (TC) levels. Results are shown as mean ± SEM. *P < 0.05 versus control group, **P < 0.01 versus control group,* $^{\#}$*P < 0.05 versus olanzapine (OLZ) 3mg/kg group,* $^{\#\#}$*P < 0.01 versus OLZ 3mg/kg group.*
