# Supplementary material for: Alterations in sorting and secretion of hepatic apoA5 induce hypertriglyceridemia due to short-term use of olanzapine
Source: Front Pharmacol. 2022 Aug 12;13:935362. doi: 10.3389/fphar.2022.935362 (PMC9411997; doi:10.3389/fphar.2022.935362)
Supplement: Supplementary file 2 [file DataSheet2.zip › Original data/Appendix.pptx]

## Slide 1
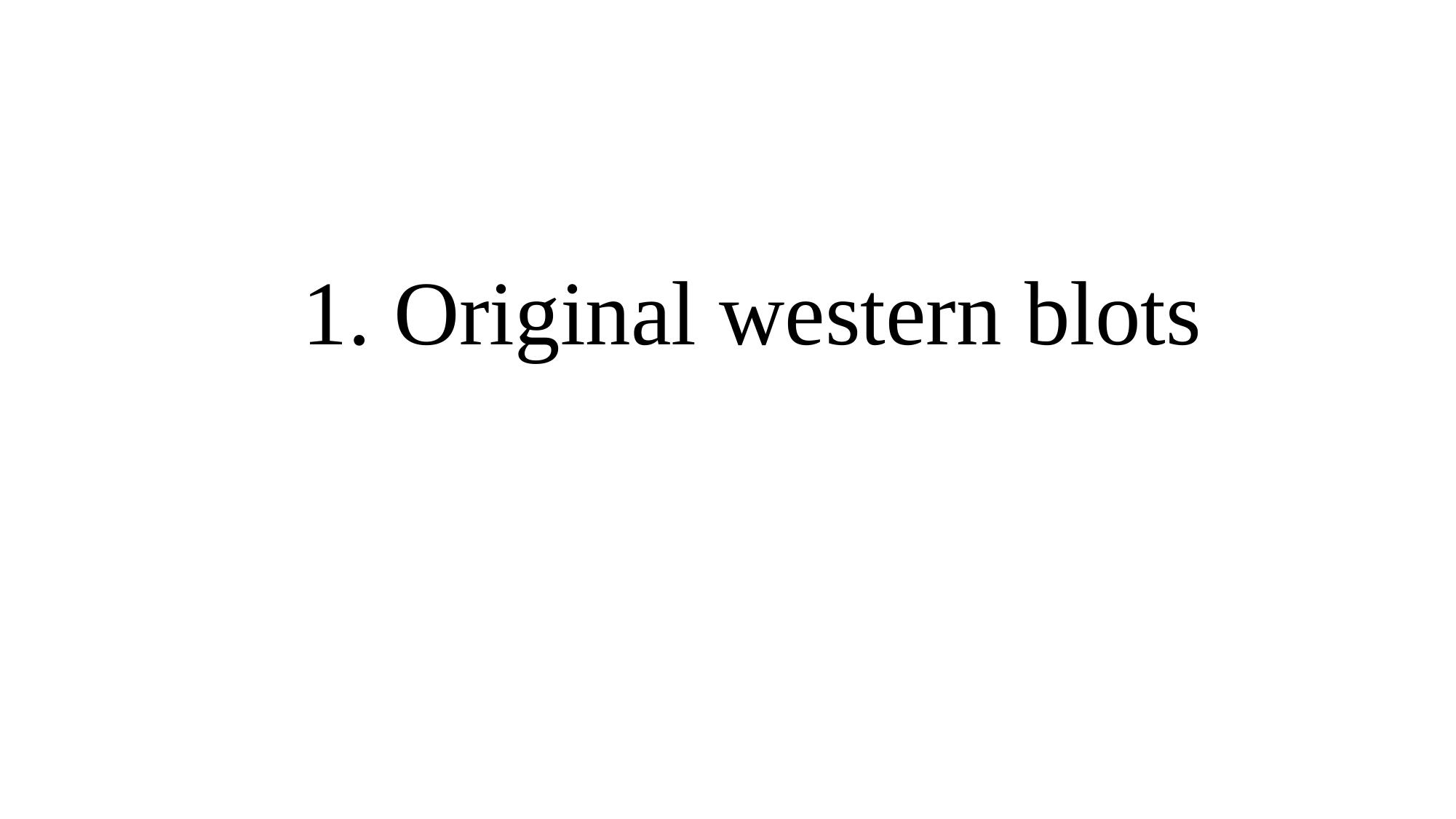

# 1. Original western blots

## Slide 2
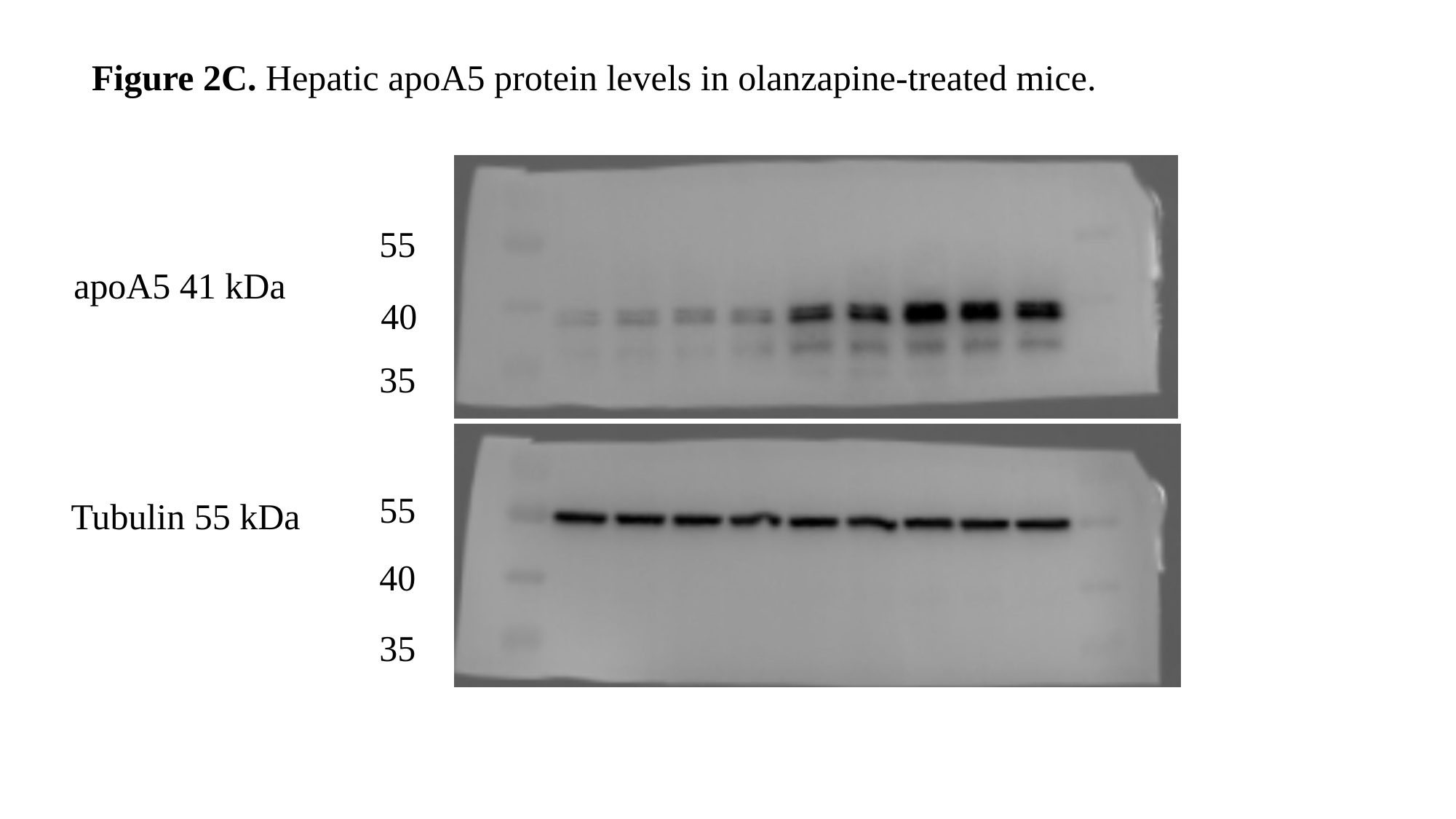

Figure 2C. Hepatic apoA5 protein levels in olanzapine-treated mice.
55
apoA5 41 kDa
40
35
55
Tubulin 55 kDa
40
35

## Slide 3
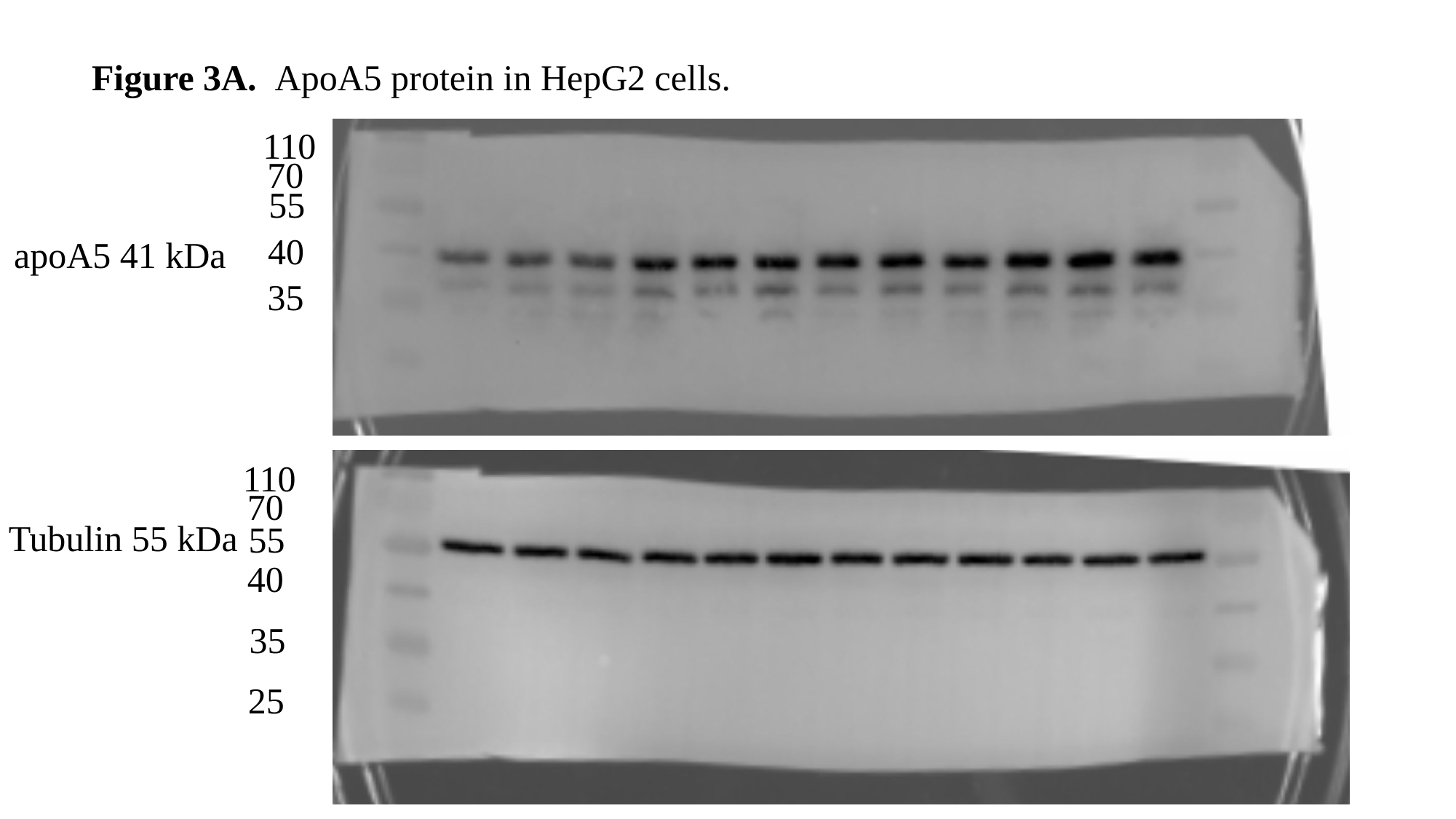

Figure 3A. ApoA5 protein in HepG2 cells.
110
70
55
40
apoA5 41 kDa
35
110
70
Tubulin 55 kDa
55
40
35
25

## Slide 4
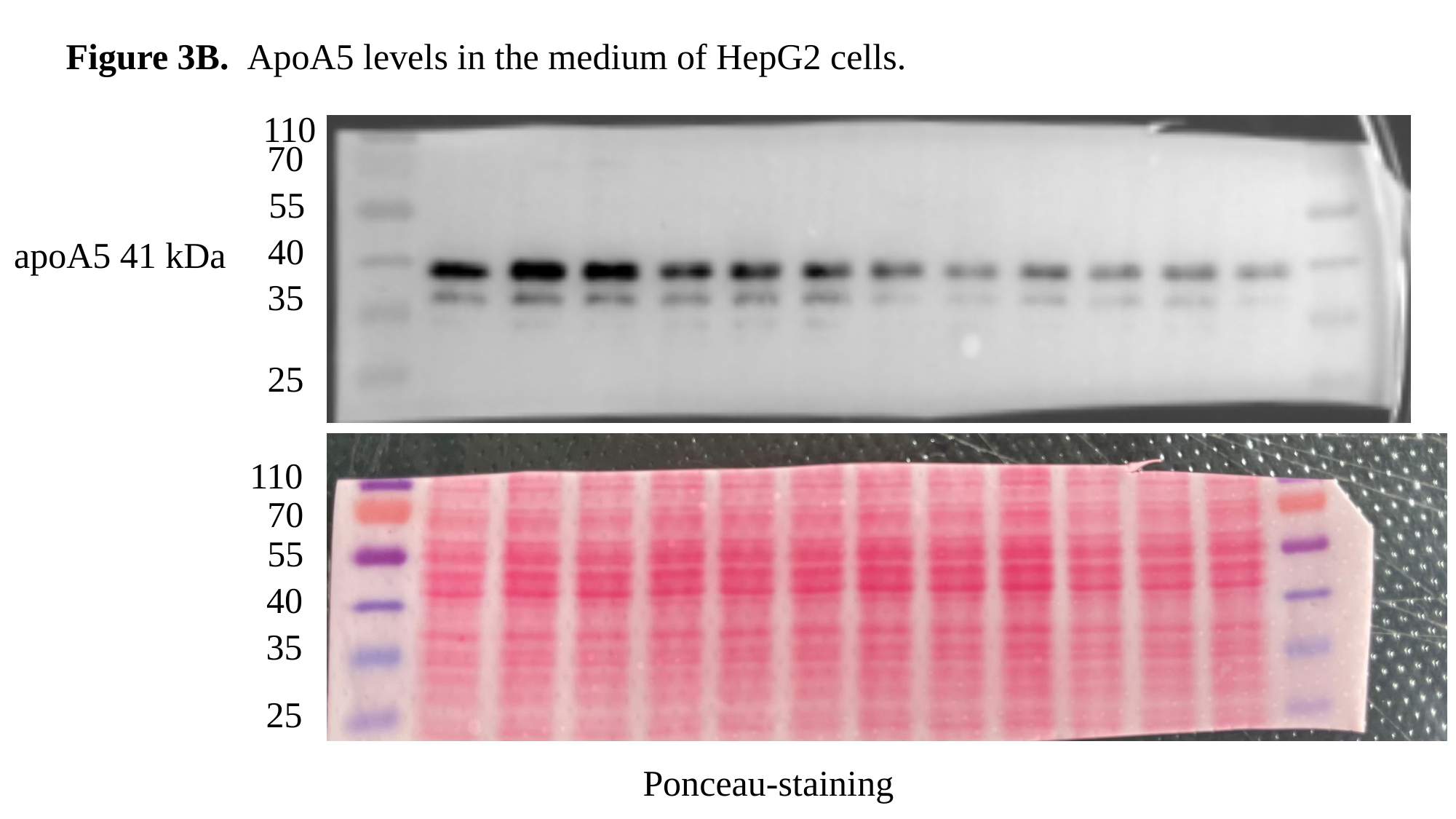

Figure 3B. ApoA5 levels in the medium of HepG2 cells.
110
70
55
40
apoA5 41 kDa
35
25
110
70
55
40
35
25
Ponceau-staining

## Slide 5
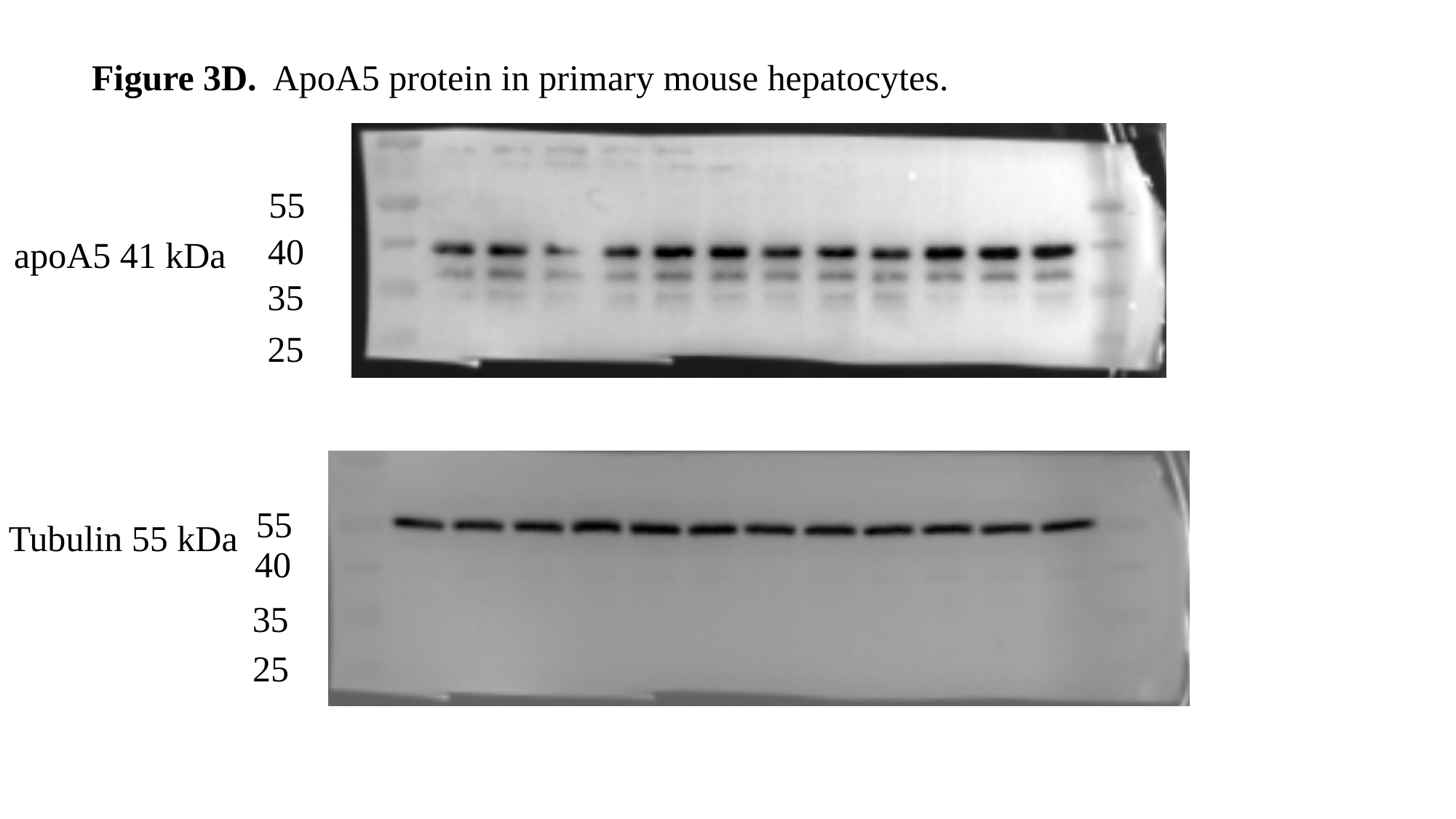

Figure 3D. ApoA5 protein in primary mouse hepatocytes.
55
40
apoA5 41 kDa
35
25
55
Tubulin 55 kDa
40
35
25

## Slide 6
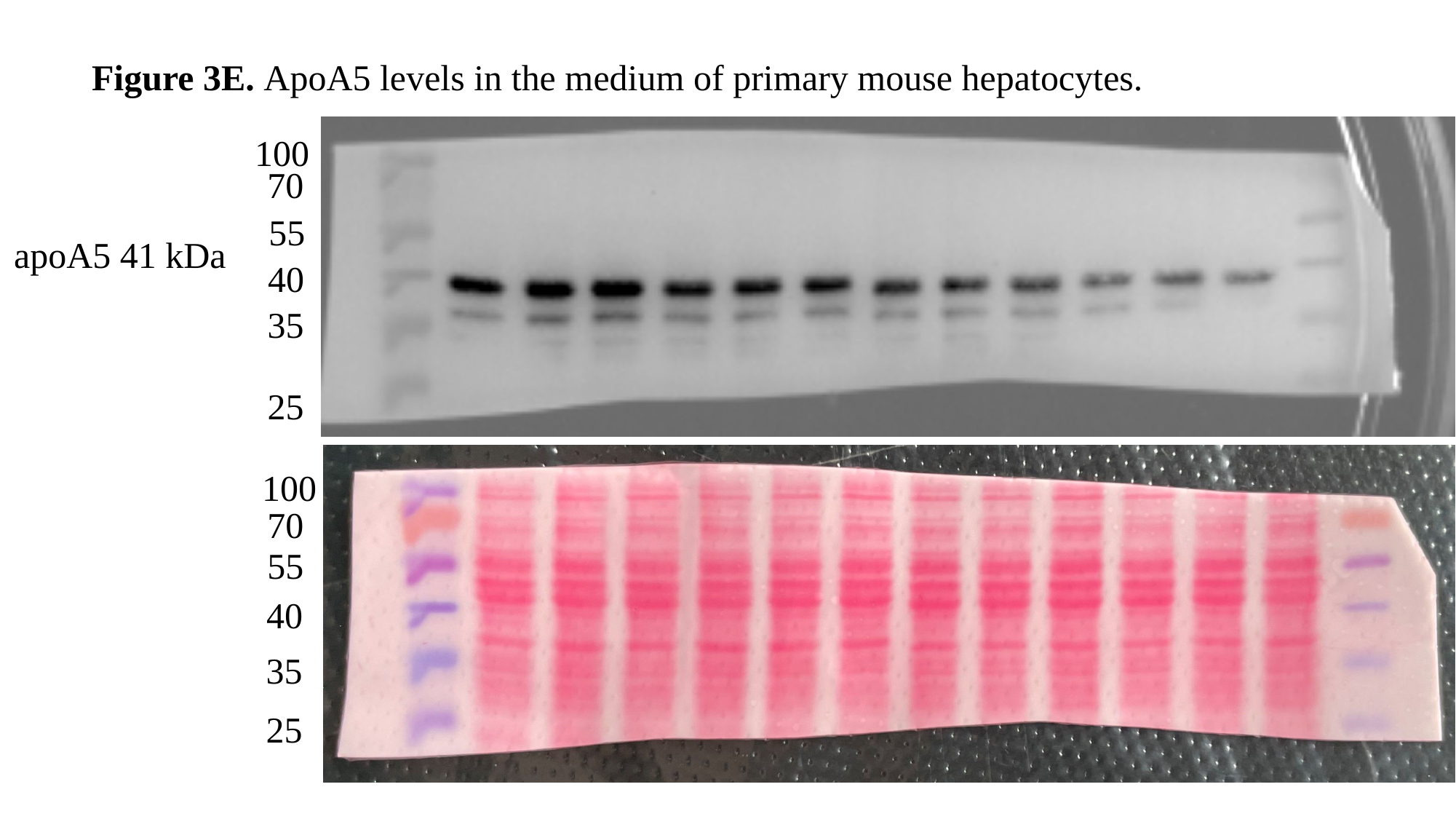

Figure 3E. ApoA5 levels in the medium of primary mouse hepatocytes.
100
70
55
apoA5 41 kDa
40
35
25
100
70
55
40
35
25

## Slide 7
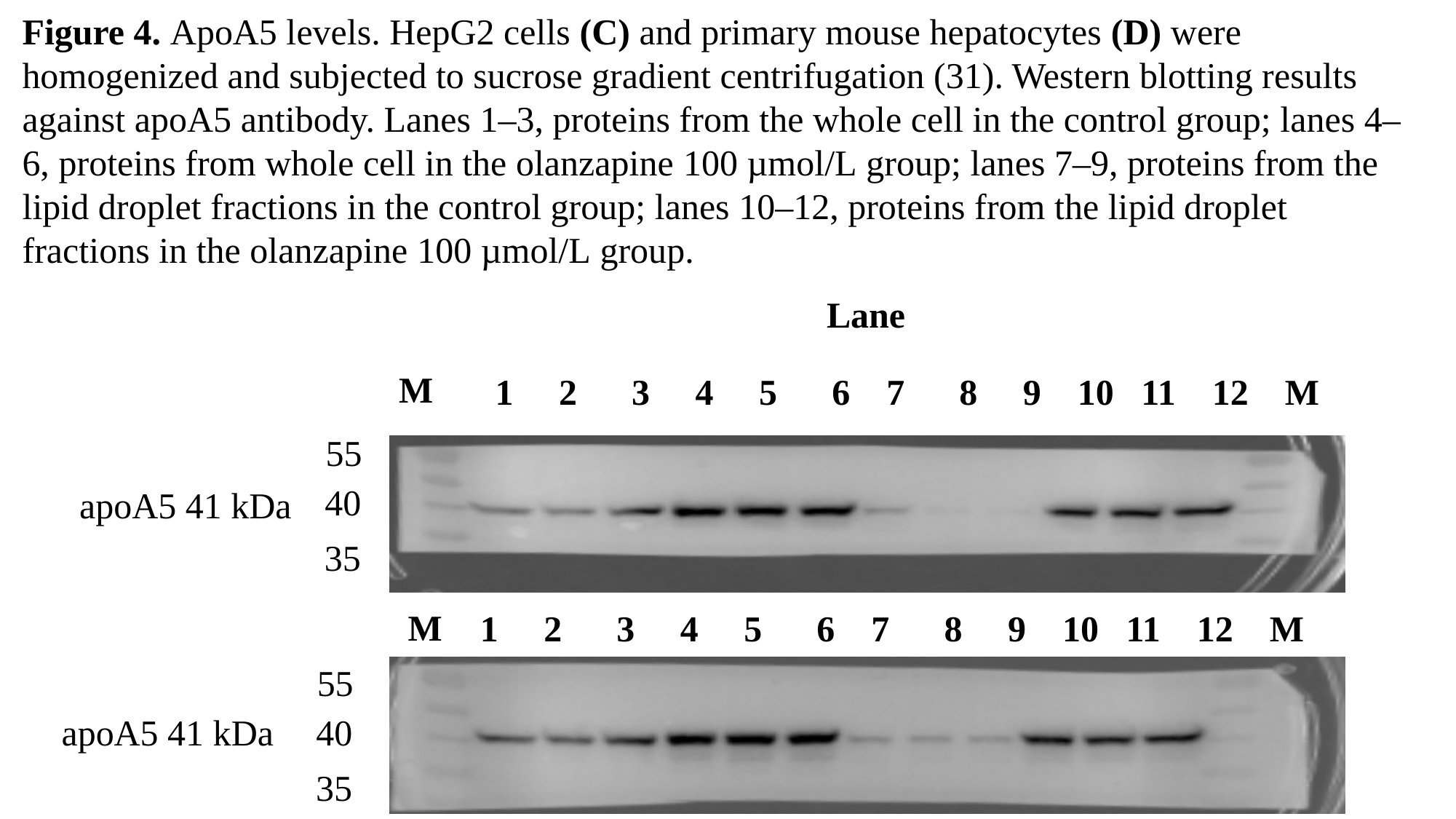

Figure 4. ApoA5 levels. HepG2 cells (C) and primary mouse hepatocytes (D) were homogenized and subjected to sucrose gradient centrifugation (31). Western blotting results against apoA5 antibody. Lanes 1–3, proteins from the whole cell in the control group; lanes 4–6, proteins from whole cell in the olanzapine 100 µmol/L group; lanes 7–9, proteins from the lipid droplet fractions in the control group; lanes 10–12, proteins from the lipid droplet fractions in the olanzapine 100 µmol/L group.
Lane
M
1 2 3 4 5 6 7 8 9 10 11 12 M
55
40
apoA5 41 kDa
35
M
1 2 3 4 5 6 7 8 9 10 11 12 M
55
apoA5 41 kDa
40
35

## Slide 8
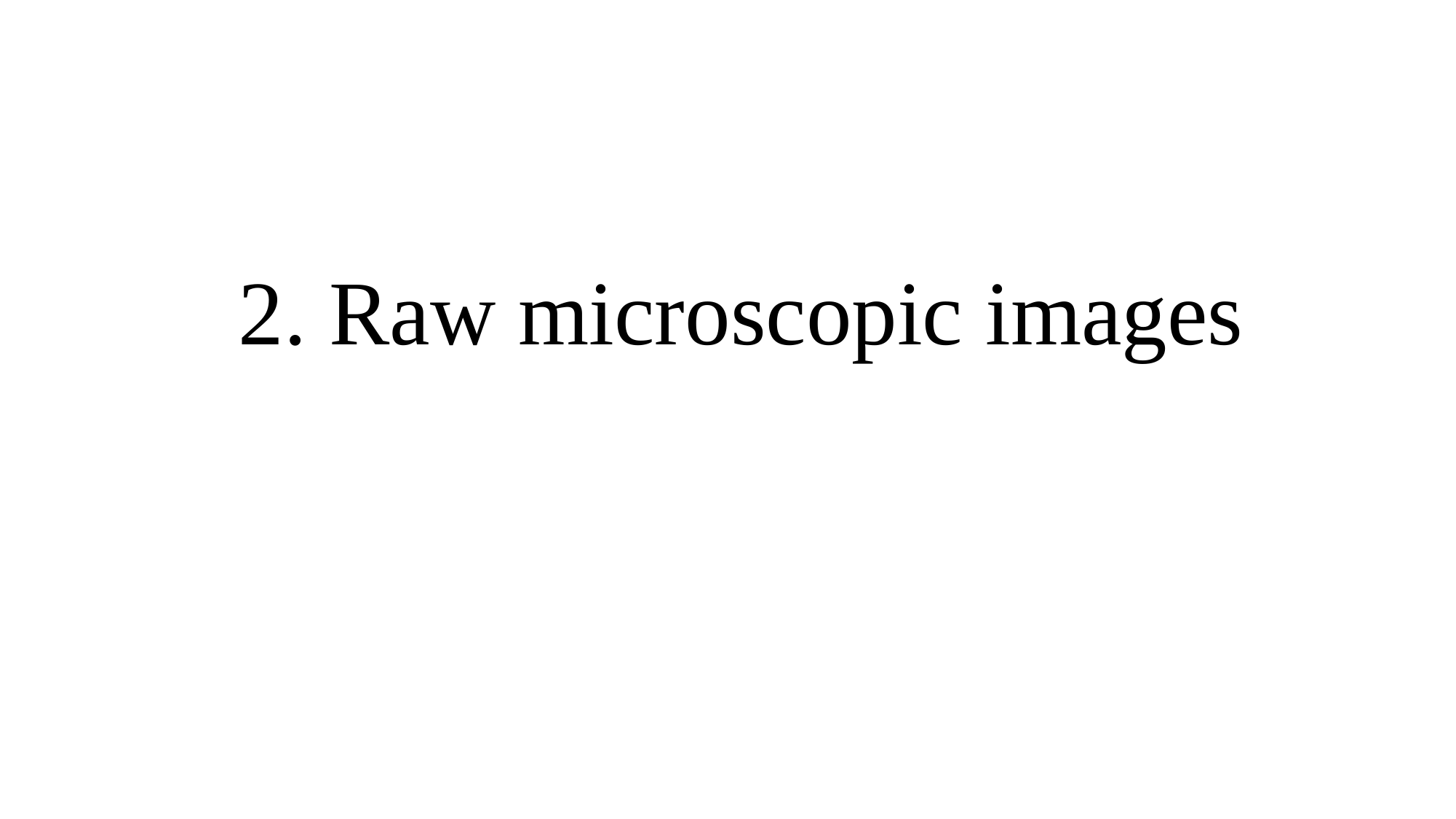

# 2. Raw microscopic images

## Slide 9
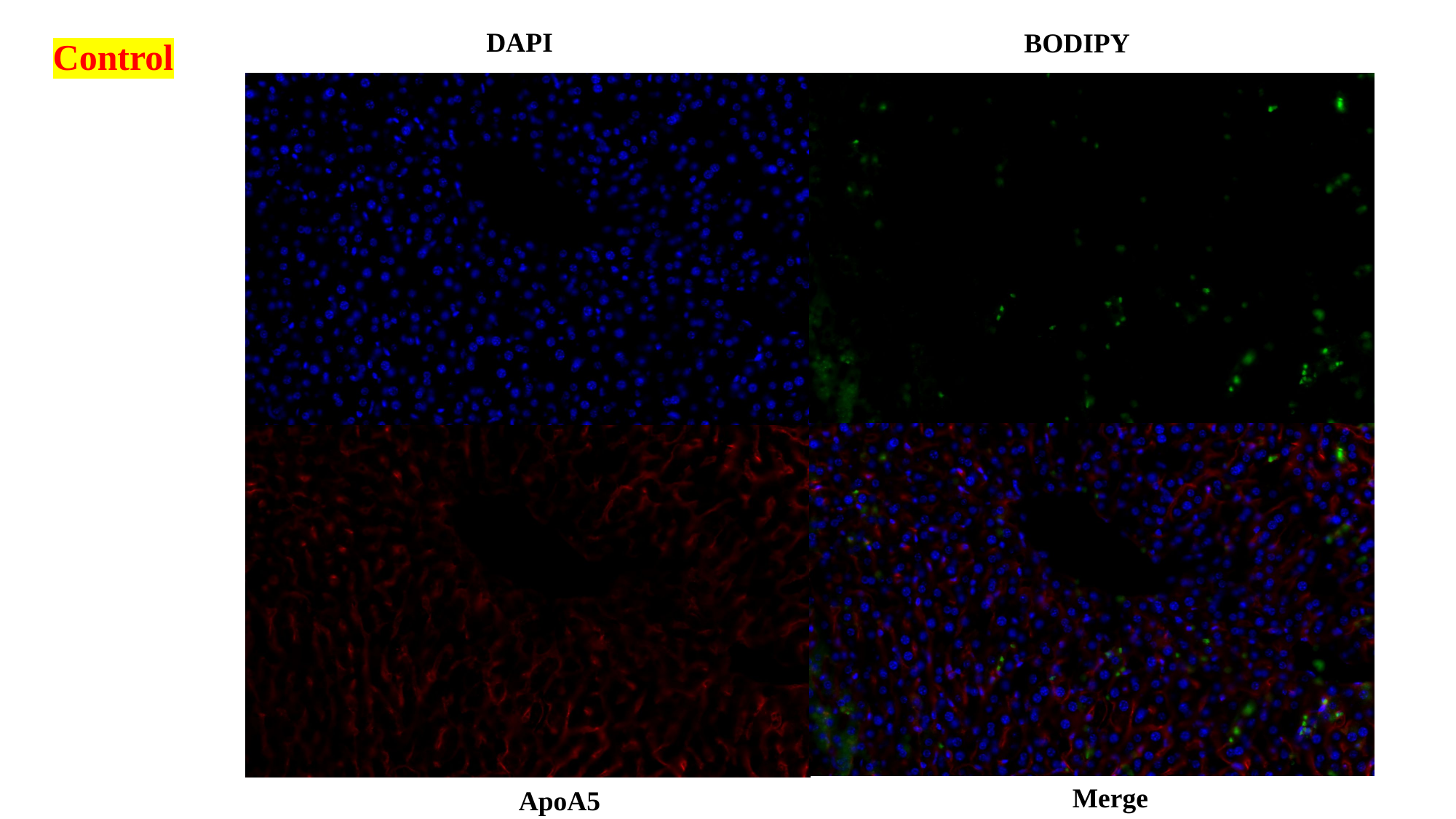

DAPI
BODIPY
Control
Merge
ApoA5

## Slide 10
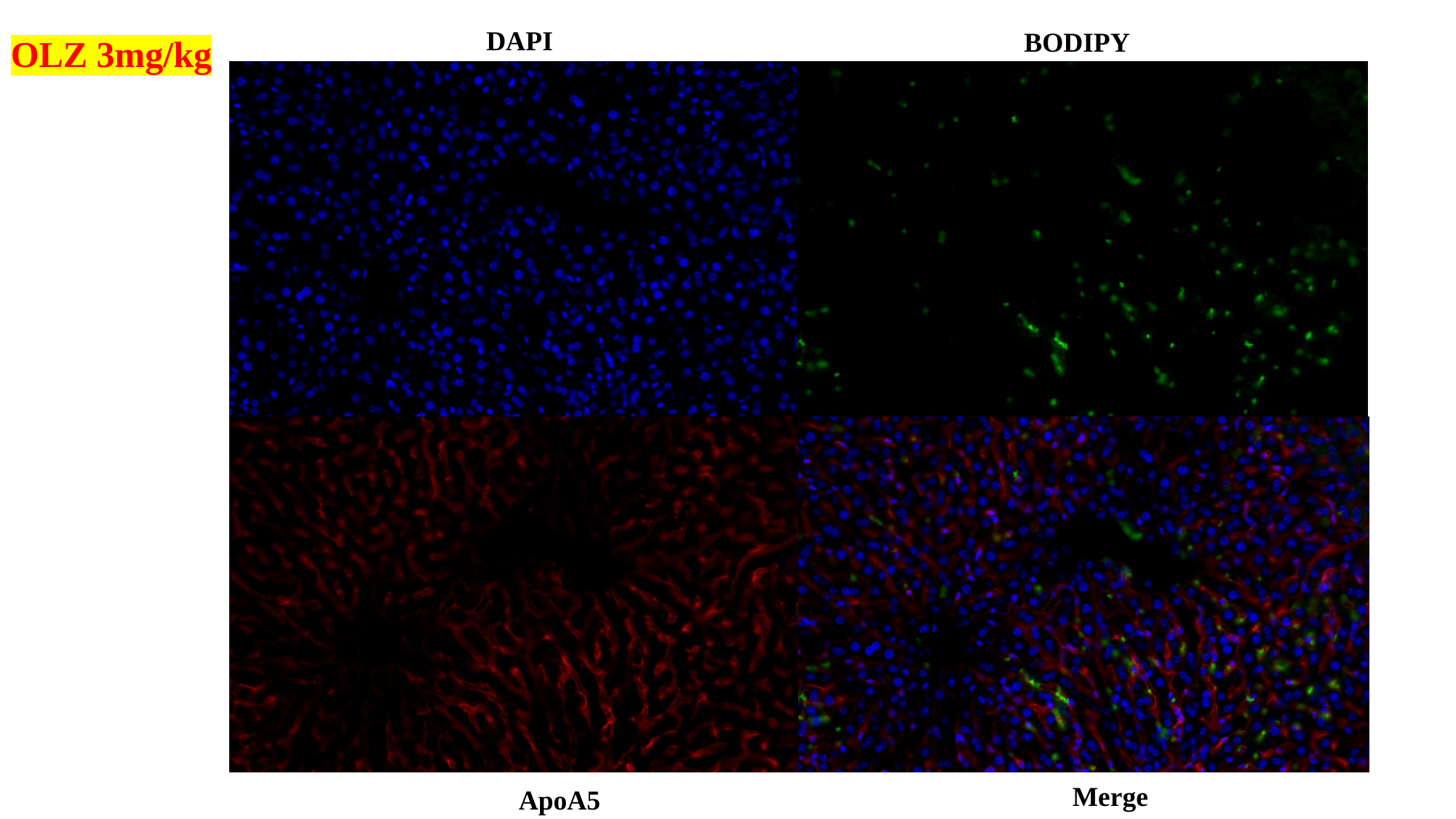

DAPI
BODIPY
OLZ 3mg/kg
Merge
ApoA5

## Slide 11
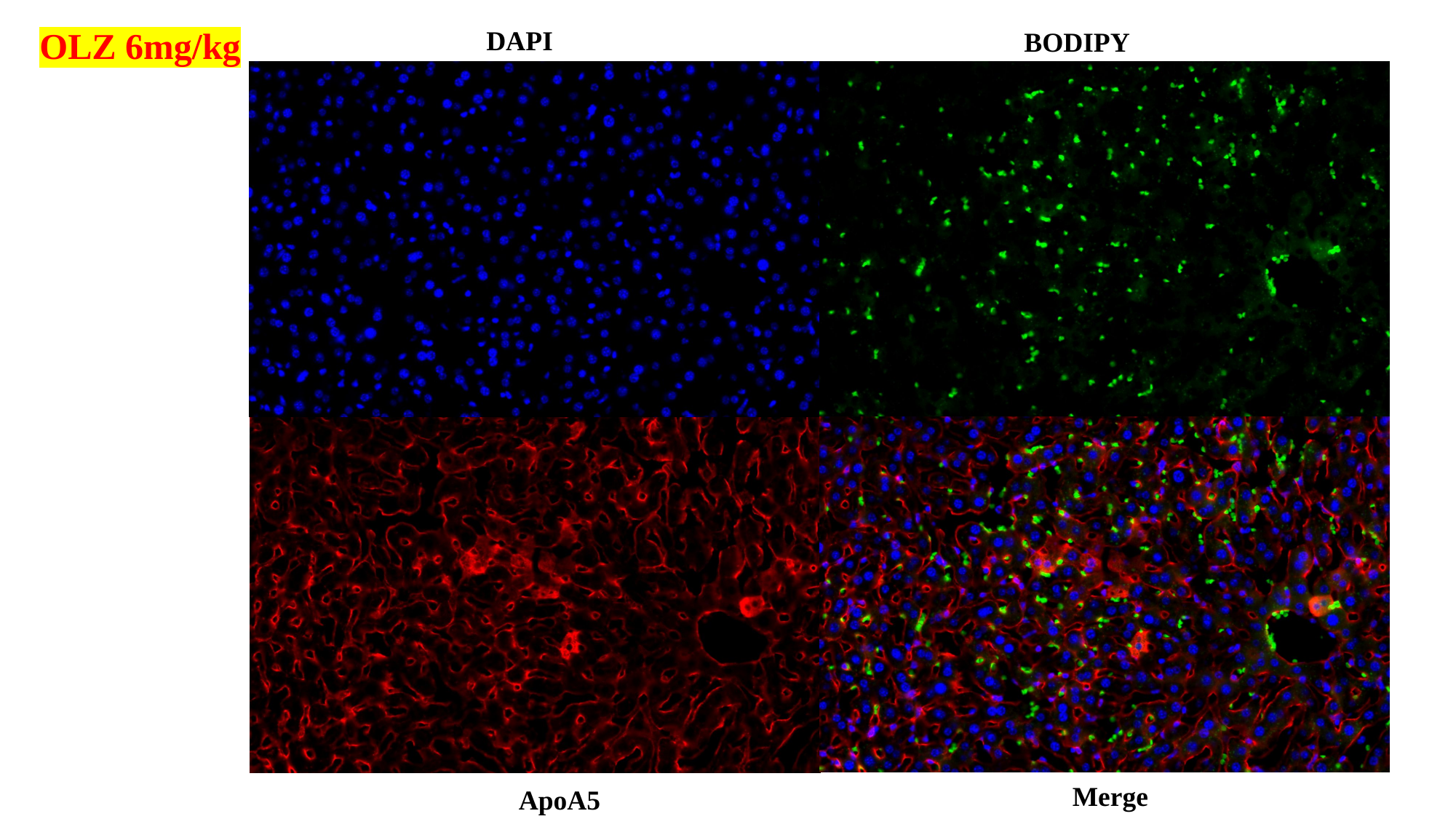

OLZ 6mg/kg
DAPI
BODIPY
Merge
ApoA5

## Slide 12
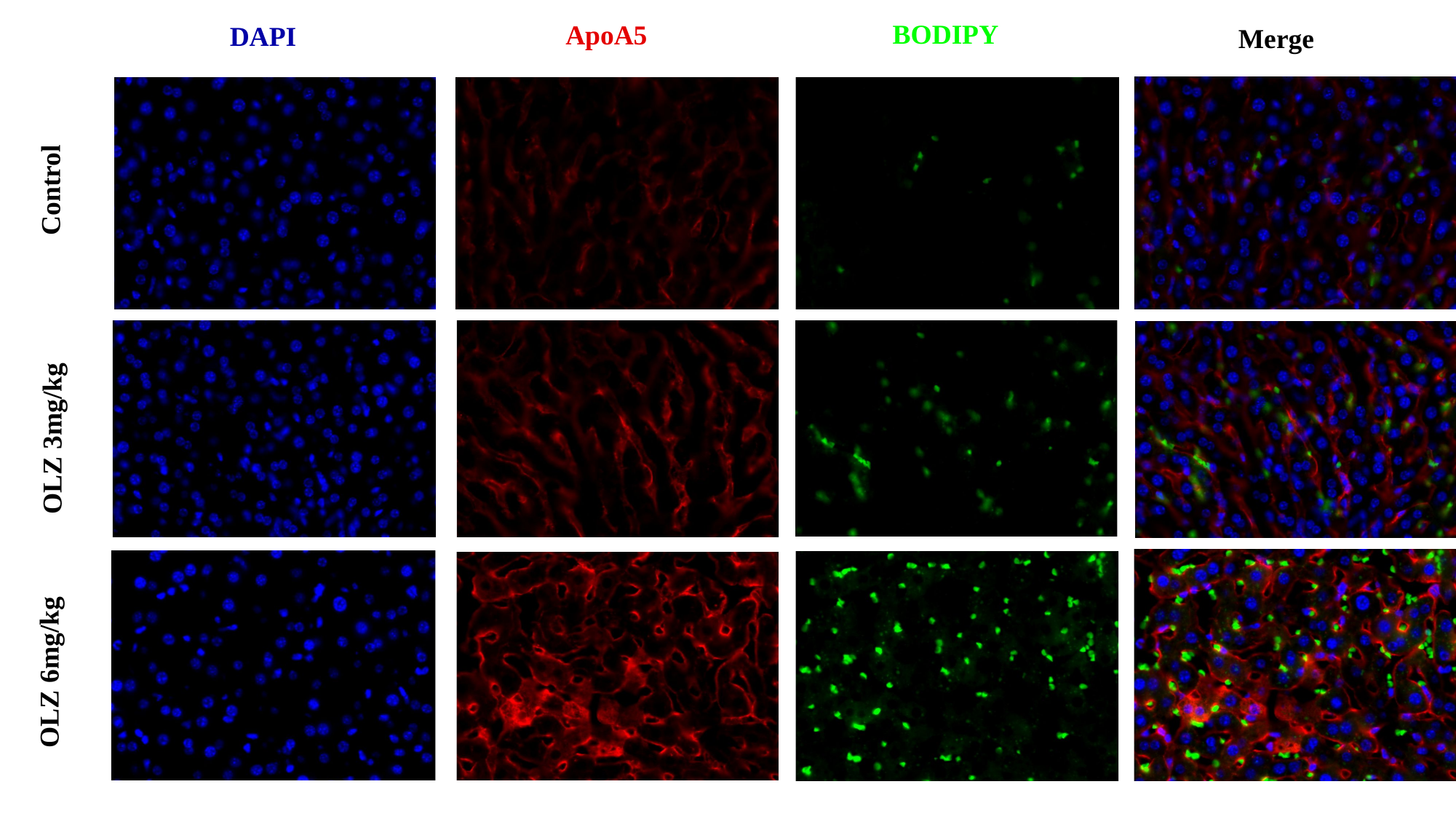

BODIPY
ApoA5
DAPI
Merge
Control
OLZ 3mg/kg
OLZ 6mg/kg
